# STHELAR, a multi-tissue dataset linking spatial transcriptomics and histology for cell type annotation

**DOI:** 10.1038/s41597-026-06937-6

**Published:** 2026-03-12

**Authors:** Félicie Giraud-Sauveur, Quentin Blampey, Hakim Benkirane, Arianna Marinello, Paul-Henry Cournède, Stergios Christodoulidis

**Affiliations:** 1 Paris-Saclay University, CentraleSupelec, Laboratory of Mathematics and Computer Science (MICS), Gif-sur-Yvette, France; 2 IHU PRISM: National Center for Precision Medicine in Oncology, Gustave Roussy, Villejuif, France; 3 Department of Medical Oncology, Gustave Roussy, International Center for Thoracic Cancers, Villejuif, France

**Keywords:** Cancer imaging, Cellular imaging, Translational research, Cancer imaging, Cancer microenvironment

## Abstract

Understanding the composition of the tumor microenvironment is critical for cancer research. Spatial transcriptomics profiles gene expressions in spatial context, revealing tissue architecture and cellular heterogeneity, but its cost and technical complexity limit adoption. To address this issue, we introduce a pipeline to build STHELAR, a large-scale dataset that integrates spatial transcriptomics with Hematoxylin and Eosin (H&E) whole-slide images for cell type annotation. The dataset comprises 31 human Xenium FFPE sections across 16 tissue types, for 22 cancerous and 9 non-cancerous patients. It contains over 11 million unique biological cells, each assigned to one of ten curated cell-type categories designed to accommodate a pan-cancer setting. Annotations were derived through Tangram-based alignment to single-cell reference atlases, followed by slide-specific clustering and differential expression analysis. Co-registered H&E images enabled the extraction of over 500,000 patches with segmentation and classification masks. Quality control steps assessed segmentation accuracy, filtered out low-confidence regions, and verified annotation integrity. STHELAR provides a reference resource for developing models to predict cell-type annotations directly from histological images.

## Background & Summary

Cancer, a leading cause of mortality worldwide with nearly 10 million deaths in 2020, represents one of the most pressing public health challenges. Defined by uncontrolled cellular proliferation and invasive characteristics, cancer includes a broad spectrum of diseases affecting various tissues and organs^1^. Modern oncology has evolved to view cancer as a complex ecosystem known as the tumor microenvironment (TME). This evolving environment comprises cancer cells and a variety of non-malignant cell types embedded in a vascularized extracellular matrix. Immune, stromal, and vascular cells within the TME significantly influence tumor progression and resistance to treatments, and the composition of this environment varies considerably depending on tumor location, primary or metastatic sites, histological subtype, molecular features, previous systemic treatment or irradiation, response to treatment, and patient-specific characteristics. Consequently, a detailed characterization of the TME is essential for the development of effective anticancer therapies^2^.

Spatial transcriptomics (ST) has emerged as a powerful tool for understanding spatial organization and cellular heterogeneity within the TME, offering insights not possible through bulk or even single-cell RNA sequencing. ST offers the unique advantage of providing spatially resolved gene expression profiles, using either imaging-based or sequencing-based approaches. Imaging-based ST relies on fluorescent labeling and signal intensity to map targeted gene expression, whereas sequencing-based methods utilize spatial barcoding coupled with next-generation sequencing to spatially quantify gene expression within tissues. Among these technologies, the Xenium platform from 10x Genomics stands out as an advanced imaging-based method, providing subcellular spatial resolution while enabling exact alignment to whole-slide images (WSIs)^3^.

Despite its potential, widespread adoption of ST technology is impeded by logistical barriers, including high cost and complex experimental protocols and data processing. In contrast, WSIs provide a more accessible resource for research by enabling the digitization of histological slides for computational analysis^4^. A WSI is a high-resolution digital scan of a tissue section, typically stained with Hematoxylin and Eosin (H&E), capturing the entire area of the slide at microscopic detail. While traditional histopathological assessment in clinical settings is still predominantly performed using optical microscopy, the use of WSIs has expanded rapidly in research, driven by advances in whole slide scanning technologies and digital pathology software^5^. Histological examination of tumor biopsy sections remains a fundamental method in oncology, offering critical insights for diagnosis and prognosis. Recent advances and growing datasets have facilitated the development of sophisticated algorithms capable of processing high-definition WSIs. Specifically, deep learning (DL) models have transformed histological image analysis workflows by achieving remarkable performance in complex clinical applications, including mitosis detection, immune infiltration quantification, cancer subtype classification, tumor grading, and prediction of gene expression profiles from WSIs^6^.

Among these tasks, cell-level instance segmentation and classification are essential for extracting interpretable and spatially resolved biological information from histological images, and they serve as a foundation for many downstream analyses. The CellViT model, a deep learning architecture based on Vision Transformers (ViT), has recently emerged as a powerful method for automated instance segmentation and annotation of cell nuclei from WSIs^7^. CellViT was trained and evaluated on the PanNuke dataset, a challenging nuclei instance segmentation dataset. This dataset consists of 7,901 H&E patches with nearly 200,000 annotated nuclei across five clinically relevant classes in 19 tissue types^8^. This model demonstrates significant accuracy in classifying nuclei into classes such as neoplastic, inflammatory, epithelial, dead, and connective cells^7^. However, these results are impeded by the limitations of the dataset the model is trained on.

The rapid progress in histological image analysis has been closely tied to the availability of high-quality annotated datasets. In particular, nucleus-level datasets have played a central role in advancing DL methods for instance segmentation and cell classification. These datasets provide the ground truth necessary to train, validate, and compare computational models at the cellular level. However, achieving a balance between annotation accuracy, cellular and tissue diversity, label granularity, and dataset scale remains a persistent challenge. A number of well-known datasets have significantly contributed to this field, each with distinct advantages and limitations. As mentioned previously, PanNuke^8^ is one of these reference datasets that offers a multi-organ scope, with a publicly accessible license. However, its annotations are restricted to coarse phenotype classifications and relatively limited cell counts. Another prominent dataset, CoNSeP^9^ consists of 41 tiles from 16 colorectal-cancer slides, encompassing approximately 25,000 manually annotated nuclei across seven categories (healthy or malignant epithelial, inflammatory, fibroblast, muscle, endothelial, and other). Although providing meticulous, gold-standard annotations, the CoNSeP dataset is highly limited by its size and single-tissue focus. Similarly, NuCLS^10^ utilizes crowdsourcing to annotate over 220,000 breast-cancer nuclei into 13 fine-grained categories (e.g., mitotic tumor, plasma cells, neutrophils), including metadata on inter-observer variability. Despite this extensive labeling, NuCLS is restricted by potential annotation noise due to its volunteer-driven approach and is limited to breast tissue. The MoNuSAC 2020 challenge dataset^11^ comprises around 46,000 nuclei from multiple tissues, validated by expert pathologists from diverse institutions to ensure staining variability. Nonetheless, the dataset excludes biologically significant cell types such as fibroblasts, endothelial cells, and plasma cells. The Lizard dataset^12^ pushes semi-automatic annotation further, providing classification for nearly 500,000 annotated colorectal nuclei into six classes with detailed immune-specific subtypes. This breadth of immune labels and half-million scale are unique for colon tissue, though reliance on model-assisted annotations risks propagating heuristic bias, and all non-colon morphologies are absent. Multi-modal strategies are now emerging, such as NeuLy-IHC^13^ that registers paired IHC and H&E sections from 19 inflammatory-bowel-disease biopsies, automatically assigning around 200,000 cells to three classes (lymphocyte, neutrophil, or other). Despite precise immune annotations, the limited cell-type diversity and tissue pathology constrain its broader application. Immunocto^14^ aligns multiplex immunofluorescence with H&E in 40 colorectal-cancer slides, annotating over 2.2 million immune cells, but limits cell annotations to immune subsets and groups all other cell types into a single residual category. It is also restricted to colon tissue. Other notable large-scale segmentation resources include the TCGA nuclei segmentation dataset, which comprises quality-controlled nuclear segmentation masks derived from more than 5,000 whole-slide images (WSIs) across 10 cancer types, with approximately five billion segmented nuclei^15^. However, this dataset does not provide any cell-type annotations. A comparative summary of the datasets is shown in Table 1. Collectively, these datasets highlight the ongoing challenge of simultaneously achieving high cell counts, diverse tissue representation, and detailed cell annotations, a critical requirement for advancing DL models. The development of such datasets is a necessary step toward improving our understanding of cancer and advancing data-driven pathology.

**Table 1:** Comparison of STHELAR with commonly used histopathology annotated datasets and the TCGA resource.

Dataset	# slides / patches	# nuclei / cells	Tissue diversity	Cell-type labels	ST / multi-omics, alignment, packaging
**STHELAR**	31 Xenium FFPE H&E WSIs / 587,555 patches at 40×/ 154,814 patches at 20x (256 × 256)	~11M unique segmented cells+nuclei	16 tissue types (22 cancer, 9 non-cancer sections, all human)	10 categories (Epithelial, Blood vessel, (Myo)Fibroblast, Myeloid, B/Plasma, T/NK, Melanocyte, Gioblastoma, Specialized, and Other) (ST-informed labels)	Xenium cell-resolved ST + explicit ST-H&E co-registration, released as SpatialData objects including multiple feature tables/embeddings (CellViT, Phikon-v2, ViT, scVI)
PanNuke	7,904 H&E patches at 40×(256 × 256)	189,744 nuclei	19 tissue types (pan-cancer)	5 categories (Neoplastic, Inflammatory, Epithelial, Dead, and Connective/Soft cells)	No ST, histology-only (patch-level)
CoNSeP	41 H&E patches (1000 × 1000) from 16 WSIs	24,319 nuclei	Colorectal adenocarcinoma	7 categories (Normal epithelial, Malignant/dysplastic epithelial, Fibroblast, Muscle, Inflammatory, Endothelial, and Miscellaneous)	No ST, histology-only (patch-level)
NuCLS	1,744 corrected patches (3,944 in total) at 40 × from TCGA FFPE WSIs of 125 patients	>220,000 nuclei (59,485 single-rater corrected, 97,000 multi-rater) / mix of polygon boundaries and boxes	Triple-negative breast cancer	13 raw categories grouped into four super-classes (Tumor, Stromal, sTILs, Other)	No ST, histology-only (patch-level)
MoNuSAC 2020	310 H&E patches at 40× sampled from WSIs of 71 patients (37 hospitals)	46,909 nuclei	4 organs (breast, kidney, lung, prostate)	5 categories (Ambiguous, Epithelial, Lymphocyte, Macrophage, and Neutrophil)	No ST, histology-only (patch-level)
Lizard (CoNIC patch release)	4,981 H&E patches at 20× (256 × 256) extracted from original Lizard dataset	495,179 nuclei	Colon tissue (aggregated from 6 sources)	6 categories (Epithelial, Lymphocyte, Plasma, Eosinophil, Neutrophil, Connective tissue)	No ST, histology-only (patch-level)
NeuLy-IHC	519 paired ROIs (H&E + IHC) for 19 H&E WSIs	235,256 cells	Inflammatory bowel disease tissue (colon/IBD context)	3 categories (Lymphocytes, Neutrophils, Other) (IHC-informed labels)	Multi-stain (H&E + IHC) but no ST, ROI-level correspondence
Immunocto	H&E patches at 40×(64 × 64) with matched multiplexed immunofluorescence (IF) from 40 patients	6,848,454 segmented cells/objects (2,282,818 immune cells)	Colorectal cancer	4 immune subtypes (CD4+ T cells, CD8+ T cells, CD20+ B cells, and CD68+/CD163+ macrophages) (IF-informed labels)	Multi-stain (H&E + matched IF) but no ST
TCGA with nuclei segmentation	5,060 WSIs with automatic nucleus segmentation +1,356 patches with manual nucleus segmentation	~5 billion nuclei	10+4 cancer types	None	No ST

**Fig. 1:**
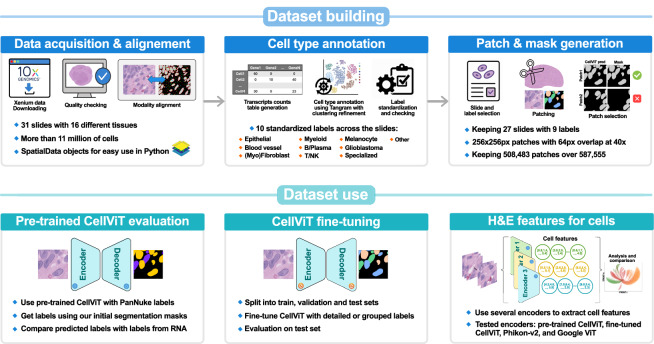
Overview of dataset construction, and downstream use case and validation. The top portion outlines the steps of dataset construction. The lower portion illustrates the use case and validation with in particular the fine-tuning of the CellViT model with the generated H&E patches and cell type masks.

The present study addresses these limitations by building a pipeline to construct an annotated dataset that leverages high-resolution ST data. The resulting dataset, named STHELAR (Spatial Transcriptomics and H&E histology for Large-scale Annotation Resource), comprises H&E-stained histological image patches with corresponding segmentation masks for nuclei, cell-type classification, and tissue provenance metadata. In addition, the dataset includes per-cell and per-nucleus RNA count matrices, standardized cell type annotations, and clustering results, all organized within fully interoperable SpatialData objects. By leveraging ST data, the method aims to generate a more comprehensive, informative, and large dataset compared to traditional annotation approaches, enabling enhanced cell-type annotation capabilities and potentially expanding coverage to a wider array of cell types. Ultimately, STHELAR could help with the development of models that predict cell types directly from routine H&E histological images. Figure 1 provides a summary of this study. More precisely, the following pipeline was followed to build this dataset:

### Building a dataset with 16 different tissues and more than 11 million unique biological cells

Data were obtained from the 10X Genomics database^16^, specifically selecting the Xenium technology that offers single-molecule detection with sub-30 nm localization resolution. This high resolution enables the spatial resolution of RNA expression at the cellular level. H&E slides were aligned with corresponding ST and DAPI data using the Xenium Explorer software^17^. Detailed characteristics of the dataset are summarized in Tables 2–3. The finalized dataset comprises 31 slides derived from 16 distinct tissues, of which 22 slides represent cancerous tissues. In total, the dataset encompasses over 11 million unique biological cells prior to patch extraction.

**Table 2:** Single-cell ST dataset with 31 slides coming from 16 different tissues (part1).

Slide ID	Tissue	Disease	Ref
bone_s0	bone	healthy	[^46^]
bone_marrow_s0	bone marrow	acute lymphoid leukemia	[^46^]
bone_marrow_s1	bone marrow	healthy	[^46^]
brain_s0	brain	brain cancer (glioblastoma multiforme)	[^47^]
breast_s0	breast	breast cancer (ductal carcinoma)	[^48^]
breast_s1	breast	breast cancer (ductal carcinoma)	[^49^]
breast_s2	same slide than breast_s0		[^50^]
breast_s3	breast	breast cancer (lobular carcinoma)	[^50^]
breast_s4	same slide than breast_s0		[^51^]
breast_s5	same slide than breast_s3		[^51^]
breast_s6	breast	breast cancer (II-A, T1cN1MX))	[^52^]
cervix_s0	cervix	cervical cancer (III-B,T1bN1MX)	[^53^]
colon_s0	image not good enough		[^54^]
colon_s1	colon	colorectal cancer adenocarcinoma	[^54^]
colon_s2	colon	colorectal cancer adenocarcinoma	[^55^]
heart_s0	heart	healthy	[^56^]
kidney_s0	kidney	healthy	[^57^]
kidney_s1	kidney	papillary renal cell carcinoma (PRCC)	[^57^]
liver_s0	liver	healthy	[^58^]
liver_s1	liver	liver cancer	[^58^]
lung_s0	alignment not good enough		[^59^]
lung_s1	lung	lung cancer (NSCLC)	[^60^]
lung_s2	same slide than lung_s3 but less relevant cell segmentation		[^61^]
lung_s3	lung	invasive acinar adenocarcinoma (IB,T2aN0MX)	[^61^]
lymph_node_s0	lymph node	reactive lymph node (left axilla)	[^62^]
ovary_s0	ovary	ovary serous carcinoma	[^63^]
ovary_s1	ovary	ovarian papillary serous carcinoma (III-B, T3bN0MX)	[^64^]
pancreatic_s0	pancreatic	pancreatic adenocarcinoma	[^65^]
pancreatic_s1	pancreatic	pancreatic ductal adenocarcinoma	[^66^]
pancreatic_s2	pancreatic	pancreatic adenocarcinoma	[^67^]
prostate_s0	prostate	prostate adenocarcinoma	[^68^]
skin_s0	image not good enough		[^69^]
skin_s1	skin	healthy	[^69^]
skin_s2	skin	melanoma	[^70^]
skin_s3	skin	melanoma	[^71^]
skin_s4	skin	primary dermal melanoma	[^72^]
tonsil_s0	tonsil	reactive follicular hyperplasia	[^73^]
tonsil_s1	tonsil	follicular lymphoid hyperplasia	[^73^]

The data were obtained from the 10× Genomics website by selecting the Xenium platform and restricting to human samples. Each slide includes three data modalities: ST, DAPI, and H&E. Gray rows denote slides excluded from the final dataset, with the reason for exclusion indicated. “Ref” links to the corresponding 10× Genomics data reference.

**Table 3:** Single-cell ST dataset with 31 slides coming from 16 different tissues (part2).

Slide ID	#Nuclei	#Genes	Panel	Seg.	#Patches (40×)
bone_s0	33,801	477	p6+custom	E	—
bone_marrow_s0	225,906	477	p6+custom	E	—
bone_marrow_s1	84,518	477	p6+custom	E	—
brain_s0	809,076	480	p4+custom	M	—
breast_s0	576,963	380	p2+custom	E	46,109
breast_s1	892,966	280	p2	E	120,000
breast_s2	same slide than breast_s0				
breast_s3	365,604	380	p2+custom	E	34,646
breast_s4	same slide than breast_s0				
breast_s5	same slide than breast_s3				
breast_s6	692,184	5,101	p8+custom	M	47,191
cervix_s0	825,663	5,101	p8+custom	M	36,585
colon_s0	image not good enough				
colon_s1	587,115	425	p3+custom	E	18,870
colon_s2	388,175	480	p4+custom	E	12,335
heart_s0	26,366	377	p6	E	5,548
kidney_s0	97,560	377	p6	E	6,948
kidney_s1	56,510	377	p6	E	4,831
liver_s0	239,271	377	p6	E	20,942
liver_s1	162,628	474	p6+custom	E	9,642
lung_s0	alignment not good enough				
lung_s1	161,000	480	p4+custom	E	11,298
lung_s2	same slide than lung_s3 but less relevant cell segmentation				
lung_s3	275,207	5,001	p8	M	20,974
lymph_node_s0	702,300	4,624	p8-dev	M	14,318
ovary_s0	247,636	480	p4+custom	E	10,230
ovary_s1	401,404	5,101	p8+custom	M	26,006
pancreatic_s0	190,965	474	p6+custom	E	26,135
pancreatic_s1	235,099	380	p4	E	12,789
pancreatic_s2	136,531	377	p6	M	7,014
prostate_s0	184,853	5,006	p8-dev	M	13,577
skin_s0	image not good enough				
skin_s1	90,106	377	p6	E	11,648
skin_s2	87,499	382	p7	E	13,234
skin_s3	106,980	282	p7	E	5,980
skin_s4	109,795	5,006	p6-dev	M	5,952
tonsil_s0	1,349,620	377	p6	E	23,402
tonsil_s1	864,388	377	p6	E	21,351

The data were obtained from the 10x Genomics website by selecting the Xenium platform and restricting to human samples. Each slide includes three data modalities: ST, DAPI, and H&E. Gray rows denote slides excluded from the final dataset, with the reason for exclusion indicated.

“#Nuclei” refers to the total number of segmented nuclei (identical to the number of cells in this context).

“#Genes” corresponds to the number of genes included in the 10× Genomics analysis panel, selected to enable the identification of major immune cell types and their subtypes.

“Panel” indicates the Xenium gene panel:

1. Pre-designed Xenium v1 Gene Expression Panels (https://www.10xgenomics.com/support/software/xenium-panel-designer/latest/tutorials/pre-designed-xenium-v1):

p1: Xenium Human Brain Gene Expression Panel

p2: Xenium Human Breast Gene Expression Panel

p3: Xenium Human Colon Gene Expression Panel

p4: Xenium Human Immuno-Oncology Profiling Panel

p5: Xenium Human Lung Gene Expression Panel

p6: Xenium Human Multi-Tissue and Cancer Panel

p7: Xenium Human Skin Gene Expression Panel

Pre-designed Xenium Prime 5K Gene Expression Panels (https://www.10xgenomics.com/support/software/xenium-panel-designer/latest/tutorials/pre-designed-xenium-prime-5k):p8: Xenium Prime 5K Human Pan-Tissue & Pathways Panel

The suffix “+custom” indicates that the panel includes a custom add-on, while “-dev” denotes a development version of the corresponding panel.

“Seg.” specifies the cell segmentation method: “E” for nuclear expansion and “M” for multimodal segmentation.

“#Patches (40×)” indicates the number of 256 × 256 patches generated at 40×, each accompanied by segmentation and classification masks. The slides bone_s0, bone_marrow_s0, bone_marrow_s1, and brain_s0 were excluded from this set due to their specific characteristics.

### Cell type annotation via Tangram with clustering refinement

Accurate cell type annotation is essential, as annotation quality substantially influences the outcomes of subsequent tasks. Several challenges arise when attempting optimal annotation using ST data. A key limitation stems from the inherently lower gene coverage of ST compared to traditional single-cell RNA-sequencing (scRNA-seq), complicating precise cell classification. Another challenge comes from the differences in gene panels, making it difficult to establish uniform, reliable annotations applicable across diverse samples.

Consequently, a slide-by-slide Leiden clustering approach was adopted. To annotate each cluster, we combined differential gene expression analysis with the Tangram method, an optimization-based probabilistic mapping approach designed to align single-cell RNA-sequencing data to spatially resolved transcriptomics measurements. Tangram learns a soft mapping between single-cell and spatial transcriptomics data using gradient-based optimization that matches spatial gene expression patterns to linear combinations of single-cell profiles, enabling robust spatial annotations across modalities^18^. Using clustering to refine the labels provided by Tangram resulted in more robust and less noisy annotations.

**Fig. 2:**
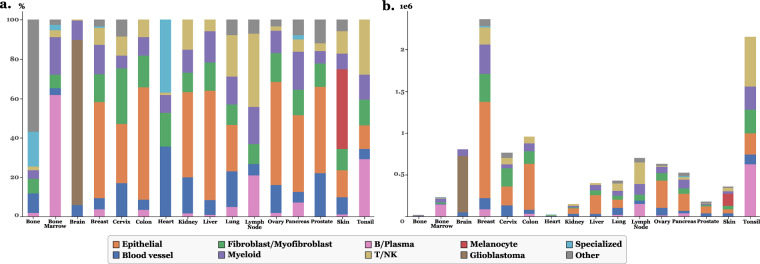
Stackplots illustrating the final dataset for all slides with final cell labels. **a**. Percentage of each cell type per tissue. **b**. Count of cell types in logarithmic scale for each tissue.

**Fig. 3:**
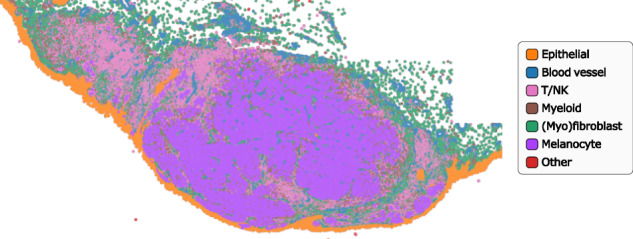
Example of annotation results at the whole-slide level, shown for the skin_s2 slide.

The final annotations were standardized across slides into the 10 following broad categories, informed by literature^2^: Epithelials, Blood vessels, Fibroblasts/Myofibroblasts, Myeloid, B/Plasma, T/NK, Melanocytes, Glioblastoma, Specialized, and Other. Table 4 provides a more detailed description of the contents of each cell category. A visual qualitative validation by a pathologist supported the biological relevance of annotations. Figure 2 shows the distribution of cell types across tissues, and Fig. 3 illustrates an example of annotation results at the whole-slide level, shown for the skin_s2 slide.

**Table 4:** Description of the content of each cell type category.

Cell type label	Description
Epithelial	Includes various epithelial cells often specific to individual tissues. For instance, in pancreatic tissue, it includes pancreatic acinar, ductal, and islet cells.
Blood vessel	Covers endothelial cells, pericytes, and smooth muscle cells.
Fibroblast/Myofibroblast	Contains fibroblasts, myofibroblasts, and mesenchymal stromal cells.
Myeloid	Comprises macrophages, monocytes, dendritic cells, neutrophils, mast cells, and plasmacytoid dendritic cells (pDCs). Due to their dual myeloid/lymphoid characteristics and rarity, pDCs were grouped here, with minimal impact expected.
B/Plasma	Combines B lymphocytes and plasma cells.
T/NK	Includes T lymphocytes and natural killer (NK) cells.
Melanocyte	Represents melanocytes or melanoma cells specifically found in skin tissue.
Glioblastoma	Identifies glioblastoma cells specific to brain tissue.
Specialized	Captures tissue-specific cells such as cardiomyocytes, osteoblasts, osteoclasts, and some endocrine cells.
Other	Encompasses cells without marker genes or those with fewer than 10 RNAs. It thus includes the final labels Unknown, Stem_like, and Less10.

### Generating H&E patches with corresponding masks

H&E image patches of size 256 × 256 px with an overlap of 64 px were extracted from each WSI at 40× resolution. Because tiling uses overlaps and because nuclei can be cut at patch boundaries, a single biological cell may contribute to multiple patch annotations. Throughout the manuscript, we refer to these duplicated occurrences as *patch-level cell instances*, in contrast to *unique biological cells*. Corresponding segmentation and classification masks were generated for each patch in alignment with the PanNuke dataset format^8^ or with the CellViT model input format^7^, using the previously established cell-type annotations at the whole cell level. To maintain the generalizability of the dataset, slides with overly specific characteristics (e.g., brain tissue or a sample from a leukemia patient) were excluded, resulting in a final dataset of 587,555 H&E patches. To support diverse research needs, patches and masks are also provided at a larger field of view with 20× resolution, resulting in a total of 154,814 patches of size 256 × 256 px.

The usefulness and relevance of this dataset were then illustrated by fine-tuning the CellViT model. Details and results are given in the technical validation section, showing its potential to bridge the gap between the rich biological insight offered by ST and the practical limitations of its broader use. By enabling the transfer of ST-derived cell type information to standard H&E images, this dataset facilitates the development of models that approximate ST insights from routine pathology slides.

## Methods

This section describes the procedure for constructing a dataset consisting of H&E image patches with corresponding masks for nucleus segmentation and cell type classification, along with tissue type annotation (i.e., breast, colon, etc.).

### ST data acquisition and processing

Single-cell ST data paired with corresponding H&E images were acquired from the 10x Genomics platform, specifically using datasets generated with Xenium technology that provides both data modalities^16^. Xenium data from the 10× Genomics dataset platform is licensed under the Creative Commons Attribution 4.0 International (CC BY 4.0) license. The data selection was restricted to human FFPE tissue sections. Manual quality inspection was performed on each slide using the Xenium Explorer software^17^ to assess tissue alignment and integrity. The software was also used to generate alignment matrices between the ST data and their associated H&E images. For each slide, we first evaluated the image-alignment file provided by 10x Genomics: when the default alignment was visually satisfactory, it was retained as-is; otherwise, alignment was refined by manually annotating corresponding keypoints across multiple distinct regions in the DAPI and H&E images, following the *Image Alignment in Xenium Explorer* tutorial^19^. Slides exhibiting substantial residual misalignment between modalities were excluded from further analysis. Among the 31 retained slides, 16 required re-alignment; these refinements used on the order of  ~30–121 keypoints per slide (median  ≈ 83, mean  ≈ 76 keypoints). Finally, the Xenium Explorer was used to extract the transformation matrix for co-registering modalities for each slide.

Subsequently, each slide was converted into a SpatialData object compatible with Python for integrated analysis of ST data, aligned H&E imagery, and segmentation information. This conversion process used foundational bioinformatics tools provided by the Scverse consortium^20^, in particular the SpatialData framework^21^, as well as functionalities available through the Sopa library^22^ (v1.1.4), facilitating direct application of the computed alignment matrix within Python. This was thus the step at which the H&E image was aligned with the ST + DAPI data using the transformation matrix constructed with the Xenium Explorer software. Existing segmentation data, specifically the DAPI-based nuclear segmentation and the default cellular segmentation, were retained along with transcript count tables detailing gene expression at the cell level. The Aggregator tool from the Sopa library was used to determine transcript counts specifically localized to the nucleus for each cell. Since cell identifiers were not consistently preserved throughout data transformations, a shape-based similarity approach for nucleus boundaries was employed to map nuclei accurately to their corresponding cells. This approach ensured the generation of a unified spatial data object encompassing separate transcript count tables for nuclei and entire cells, as well as coherent and unique cell identifiers aligning nuclei and cellular boundaries. The final composition of the dataset is summarized in Tables 2–3.

The 10x Genomics Xenium dataset pages did not provide an explicit patient identifier for each slide. The available metadata included (i) the dataset web-page URL (which grouped one or several sections released together) and (ii) the “donor count” reported on that page. When a given URL contained *n* slides and reported a donor count of *n*, we conservatively assumed that each slide originated from a different donor. Across different dataset URLs, we also assumed slides came from different donors, except in the specific situation where visual inspection of the H&E whole-slide image indicated that two releases corresponded to the exact same physical tissue section that was processed again (e.g., re-run with a different gene panel). In such cases, only one instance was retained, and the duplicates were excluded (Tables 2–3). Under these assumptions and filtering steps, all slides retained in STHELAR correspond to distinct patients (i.e., one slide per patient). Therefore, the number of patients contributing to the STHELAR dataset is equal to the number of retained slides listed in Tables 2–3. Cancer versus non-cancer status is provided at the slide level (22 cancer and 9 non-cancer sections) in this Table 2.

### Cell type annotation

Cell type annotation posed significant challenges due to the limited number of assayed genes and heterogeneous gene panels across slides. Multiple approaches were initially explored to overcome these obstacles and achieve robust cell type annotation with consistent and generalizable cell type categories. Early attempts involving batch-corrected clustering across all slides using methods such as Harmony^23^, scVI embeddings^24^, and pre-trained embeddings from scGPT, a foundation model trained on extensive single-cell datasets^25^, failed to yield conclusive results largely due to persistent batch effects. The final choice was to use Tangram annotations combined with cluster-based refinement per slide. Other spatially informed clustering methods have been developed for ST data to integrate spatial coordinates with gene expression, often improving spatial domain delineation compared to expression-only clustering. However, these methods are typically designed to identify spatially contiguous domains, whereas our objective is high-precision cell-type labels at single-cell resolution across heterogeneous tissues and gene panels. In this setting, strong spatial smoothness assumptions may reduce cell-type purity due to local mixing. This is why we used expression-based clustering per slide as a curation scaffold, with final labels determined by marker-gene evidence. The annotation process was conducted twice: first using the *nucleus RNA count matrices* and then using the *whole-cell RNA count matrices*.

#### Tangram reference

Tangram, a method that learns spatial alignment between sc/snRNA-seq data and a reference spatial dataset, was used for initial cell type annotation^18^. The selection of an appropriate reference dataset has a significant impact on annotation accuracy. Three reference sources were evaluated: CellXGene^26^, Curated Cancer Cell Atlas^27^, and the Deeply Integrated human Single-Cell Omics (DISCO)^28^ platform datasets. Quantitative assessments demonstrated that DISCO provided more comprehensive and representative cell types, making it the preferred choice. In practice, we used tissue-matched single-cell reference atlases for Tangram-based label transfer. For nearly all slides, the reference was a DISCO tissue sub-atlas downloaded from the DISCO portal (https://disco.bii.a-star.edu.sg/download), using the exact subset name and version reported on the portal (Table 5). DISCO datasets are uniformly pre-processed by the DISCO consortium from raw sequencing reads using a standardized pipeline^28^. For the prostate slide only (prostate_s0), where no DISCO reference existed, we instead used the Curated Cancer Cell Atlas (3CA) prostate dataset from Song *et al*. 2022 as provided on the 3CA portal (https://www.weizmann.ac.il/sites/3CA/)^29,30^. All reference files were downloaded and passed directly, without further modification, to the Sopa library^22^, which handles the Tangram workflow. Importantly, no expression imputation was performed for genes absent from Xenium.

**Table 5:** Reference single-cell atlases used for Tangram label transfer.

Source	Subset	Version	STHELAR slide IDs
DISCO	bone marrow	2.0	lymph_node_s0, bone_marrow_s0, bone_marrow_s1, bone_s0
DISCO	brain	1.0	brain_s0
DISCO	breast	2.1	breast_s0, breast_s1, breast_s3, breast_s6
DISCO	heart	1.0	heart_s0
DISCO	intestine	1.0	colon_s1, colon_s2
DISCO	kidney	1.0	kidney_s0, kidney_s1
DISCO	liver	2.0	liver_s0, liver_s1
DISCO	lung	2.0	lung_s1, lung_s3
DISCO	ovary	1.0	ovary_s0, ovary_s1, cervix_s0
DISCO	pancreatic	1.0	pancreatic_s0, pancreatic_s1, pancreatic_s2
DISCO	skin	1.0	skin_s1, skin_s2, skin_s3, skin_s4
DISCO	tonsil	1.0	tonsil_s0, tonsil_s1
3CA	prostate	Song *et al*. 2022	prostate_s0

For each Xenium slide, Tangram alignment was performed against the tissue-matched DISCO sub-atlas (subset name and version as reported by the DISCO download portal) or, for prostate, the 3CA prostate dataset (Song *et al*. 2022).

Tangram was executed using only the intersection of genes between each Xenium panel and its corresponding reference atlas, which in practice corresponds to the Xenium gene set, as ST panels contain far fewer genes than single-cell RNA-seq references.

#### Tangram mapping via Sopa

We transferred cell-type labels from tissue-matched single-cell atlases to Xenium single-cell data using Tangram^18^ through the sopa.tangram_annotate wrapper from Sopa (v1.1.4) calling tangram-sc (v1.0.4)^22^. Sopa ran Tangram in cell-to-cell mode (mode=“cells”), which learns a probabilistic mapping matrix between individual reference cells and individual spatial observations. In this setting, Tangram is used as a probabilistic label-transfer prior: each Xenium cell is modeled as a soft linear combination of reference single-cell expression profiles over the shared genes, and annotations are projected from the reference onto Xenium using tangram.project_cell_annotations. Importantly, Tangram’s optimization objective is not restricted to spot-based assays, it only requires a spatial expression matrix and a single-cell reference on a shared gene set. Thus, when the spatial measurement unit is already a segmented cell (Xenium), the method naturally operates at single-cell spatial resolution and is interpreted as probabilistic label transfer rather than spot deconvolution. Finally, to reduce sensitivity to any residual mismatch between scRNA-seq references and targeted Xenium panels, we used Tangram strictly as an initial prior. As detailed later, predictions were summarized at the Leiden-cluster level, and the final labels were assigned primarily from within-slide marker-gene evidence, with Tangram used only as supporting context. Tangram was run using only the intersection of genes between each Xenium panel and the corresponding reference atlas (no imputation was performed for genes absent from Xenium). For each slide (and each bag; see below), Sopa performed Tangram-compatible preprocessing on the Xenium AnnData: it used raw counts, removed genes with zero counts, aligned gene symbols case-insensitively between Xenium and the reference, and restricted training to genes expressed in both datasets. No spatial smoothing or neighborhood-based post-processing of Tangram predictions was applied. Sopa enabled density correction using the Tangram v1.0.4 default rna_count_based density prior. In Tangram, providing a density prior activates the density regularization term by setting lambda_d=1. Sopa used the default Tangram optimization settings and loss weights (num_epochs=1000, learning_rate=0.1, lambda_g1=1, lambda_g2=0, lambda_r=0). Here, lambda_g1 weights the main gene-expression reconstruction term that drives alignment, lambda_g2 (disabled) would weight an auxiliary voxel-gene term, and lambda_r (disabled) would apply an entropy regularizer on mapping probabilities. Constrained mapping (mode=“constrained") was not used, thus no filter vector (F_out) and no target_count/lambda_count/lambda_f_reg terms were used, and no explicit Tangram-based doublet filtering was performed. To reduce RAM/VRAM usage on large slides, Sopa executed Tangram on random bags of Xenium cells of size 10,000 ($\lceil {n}_{{\rm{cells}}}/10,000\rceil$ splits per slide). At each annotation level, the reference atlas was capped at max_obs_reference=10,000 cells by random subsampling when needed. Tangram predictions were computed per bag and merged back into a full-slide probability table. Sopa then produced hard labels from per-class probabilities by clipping extreme values (default clip percentile 0.95), applying per-class min-max scaling, and assigning the argmax label per Xenium cell.

#### Tangram refinement via Leiden clustering and gene differential analysis

**Fig. 4:**
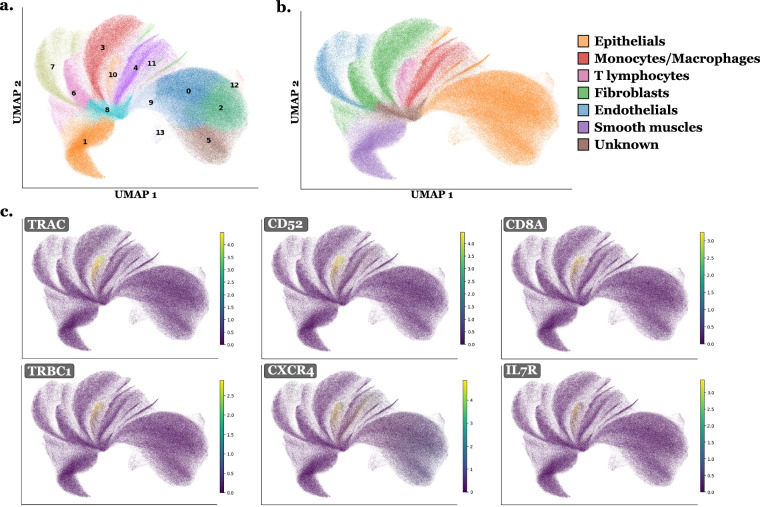
UMAPs illustrating the cell type annotation process. This is an example for the ovary_s1 slide with Leiden clustering at resolution 0.4, using *whole-cell RNA count matrice*. In **a**. cells are labeled by their cluster number. Differential expression analysis was performed on these clusters and marker genes are analyzed for each, as illustrated in **c**. with examples of marker genes for cluster 10. In **b**., cells are labeled with their final labels for the slide, combining information from both marker genes and Tangram labels.

The Tangram annotations are quite noisy and overly precise relative to the level of detail achievable with the ST data, and they depend strongly on the quality of the reference, as illustrated in Fig. 8. To address this issue, we refined the Tangram annotations by performing slide-specific clustering (using scanpy v1.10.2). Cells (or nuclei, depending on the RNA compartment under consideration) with fewer than 10 transcripts were filtered out. Normalization was conducted to equalize transcript counts across cells, followed by log-transformation. Unit variance scaling was applied to each gene, and Principal Component Analysis (PCA) was performed for dimensionality reduction and noise removal. A neighborhood graph was constructed using the first principal components and 10 nearest neighbors. Clustering was conducted using the Leiden algorithm at several resolutions (mainly 0.2, 0.4, and 0.6), with Uniform Manifold Approximation and Projection (UMAP) applied for visualizing clusters. A final clustering granularity was manually selected per slide based on UMAP structure and cluster separability. Additional sub-clusters were optionally retained when the UMAP suggested biologically meaningful heterogeneity. Table 6 reports, for each slide, the number of clusters obtained at each tested Leiden resolution, the number of clusters ultimately selected for the differential gene expression analysis, and the final number of high-level labels assigned after the analysis, using the clustering from *whole-cell RNA count matrices*. Marker genes for each retained final cluster were identified using differential gene expression analysis (Wilcoxon signed-rank test with Benjamini-Hochberg correction). Final cell types were assigned by integrating Tangram annotations, UMAP visualizations of marker gene expression across clusters, and literature-based biological context. More precisely, for each cluster, marker genes were systematically inspected to determine its dominant biological identity. In parallel, Tangram labels were summarized at the cluster level by reporting the top seven most frequent Tangram cell types and their relative proportions within the cluster. Because Tangram labels are highly fine-grained and depend strongly on the reference atlas used, they were used strictly as a guideline to inform manual curation rather than as an absolute decision rule. The final label assigned to each cluster was determined primarily by marker-gene evidence. In cases of agreement between marker genes and Tangram, the label was directly validated. In cases of discordance, the marker genes took precedence over Tangram predictions, as differential expression reflects transcriptomic evidence intrinsic to the slide itself, whereas Tangram predictions depend on the external reference atlas. This procedure resulted in two intermediate annotation levels per slide, with a higher-level set of labels and a lower-level refined set, both defined prior to cross-slide standardization. In practice, Tangram was run exclusively on *nucleus RNA count matrices*, as these were the most robustly defined transcriptomic compartments across all slides given the variability in cell boundary estimation. To reduce computational burden, Tangram was not re-run on whole-cell RNA matrices. Instead, for each cell, the final nucleus-derived Tangram label was propagated to the corresponding full cell as the initial guideline. Figure 4 illustrates the annotation process using the ovary_s1 slide as an example. Anndata^31^, Scanpy^32^, and Rapids-singlecell^33^ toolkit were used to perform these analyses using GPU acceleration. These final labels were then generalized and standardized across slides into the following categories: Epithelial, Blood_vessel, Fibroblast_Myofibroblast, Myeloid, B_Plasma, T_NK, Melanocyte, Glioblastoma, Specialized, Stem_like (potential stem cells, no clear marker genes), and Unknown (cells lacking specific gene markers). This categorization aimed to balance annotation accuracy, biological relevance, and consistency across tissue types. For cells or nuclei containing fewer than 10 RNAs, which were excluded from the analysis, the label Less10 was assigned. Subsequently, the final labels Unknown, Stem_like, and Less10 were usually merged into a single category Other (Table 4). For all subsequent analyses and dataset building, cell-type annotations based on *whole-cell RNA count matrices* were used.

**Table 6:** Per-slide Leiden cluster counts across tested resolutions and final retained label granularity, using clustering from whole-cell RNA count matrices.

SlideID	res0.2	res0.4	res0.6	res0.8	Final #clusters	Final #classes
bone_s0	6	7	11	—	11	9
bone_marrow_s0	5	10	13	—	10	7
bone_marrow_s1	5	8	12	—	12	9
brain_s0	6	9	11	—	9	5
breast_s0	9	15	18	—	11	9
breast_s1	6	10	13	—	10	6
breast_s3	9	16	17	—	16	9
breast_s6	7	10	12	—	12	9
cervix_s0	8	11	15	—	15	7
colon_s1	7	9	13	—	13	7
colon_s2	5	9	14	—	14	7
heart_s0	8	11	13	—	12	9
kidney_s0	9	13	16	—	13	9
kidney_s1	8	10	13	—	13	10
liver_s0	7	11	16	—	11	5
liver_s1	6	11	13	16	12	7
lung_s1	7	11	14	—	12	10
lung_s3	14	17	21	—	16	11
lymph_node_s0	10	14	17	—	14	12
ovary_s0	4	9	12	—	10	7
ovary_s1	9	13	18	—	14	7
pancreatic_s0	7	11	16	—	12	8
pancreatic_s1	5	10	13	17	14	10
pancreatic_s2	9	10	14	—	14	8
prostate_s0	8	12	14	—	14	9
skin_s1	15	22	22	—	17	7
skin_s2	5	10	14	—	10	6
skin_s3	4	9	14	18	12	6
skin_s4	11	13	19	—	12	10
tonsil_s0	4	9	14	—	14	8
tonsil_s1	7	11	13	17	16	10

Columns “res0.2–0.8” report the number of Leiden clusters obtained on each slide at the corresponding resolution. “Final #clusters” corresponds to the number of clusters used for the differential gene expression analysis and deep review. “Final #classes” refers to the number of curated cell-type classes at high level after Tangram + marker-based refinement.

Figure 8 illustrates the comparison between Tangram labels and the final labels derived from the nucleus RNA count matrices. To facilitate comparison between the two annotation schemes, Tangram labels were manually grouped into the corresponding final label categories. The correspondence between the two is not perfect, as Tangram was primarily used as a guideline rather than as a definitive annotation method. The UMAPs for the lung_s3 slide provide a visual example of concordance between the two approaches and highlight a cluster in which the dominant Tangram label does not match the final label. Marker gene expression is shown to justify the choice of the final label. This example demonstrates the value of the proposed approach, which leverages Tangram as a guiding tool rather than as the final decision-making method.

One limitation encountered during annotation was differentiating normal from cancerous cells, as RNA analysis alone did not yield clear distinctions, especially due to the continuous nature of gene expression profiles rather than discrete differences between normal and cancerous cells. Several approaches were explored, including pathway analyses such as KEGG pathway^34^, which offers a collection of manually drawn pathway maps representing the knowledge of the molecular interaction, reaction, and relation networks for different types of cancers. However, this yielded inconclusive results, and annotations for these categories were retained but marked with limited confidence and not used afterward. A similar challenge arose when attempting to identify dead cells. While low RNA content could serve as a proxy indicator for cell death, it does not conclusively confirm the status. However, qualitative assessment by the pathologist supported this interpretation, as many low-RNA cells exhibited morphological features consistent with cell death. As a provisional measure, these low-RNA cells (Less10) were grouped within the Other category, alongside cells previously categorized as Unknown or Stem_like. Both tumor and dead cells represent highly heterogeneous categories, making them particularly difficult to annotate consistently and challenging to predict.

#### Assessment of within-tissue batch effects across slides with different Xenium panels

**Fig. 5:**
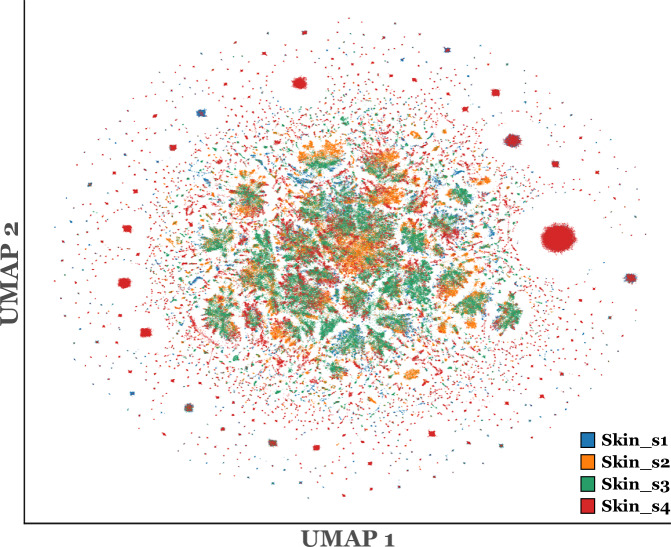
Within-tissue cross-slide integration in skin slides. UMAP obtained by jointly preprocessing and clustering the four skin slides retained in STHELAR (skin_s1 - skin_s4) after restricting expression to the 47 genes common to all four slides. Cells are colored by slide identifier. The Leiden clustering at resolution 0.4 outputs 1,291 clusters.

To explicitly evaluate whether batch effects were present across slides of the same tissue acquired with different Xenium gene panels, we performed a joint clustering analysis on the four skin slides retained in STHELAR (skin_s1 - skin_s4), which span different panels (Table 3). We restricted the expression matrices to the intersection of genes across these slides, which comprised only 47 genes. Using this reduced gene set, we applied the same preprocessing and graph-based workflow as in our refinement step (filtering cells with fewer than 10 transcripts, library-size normalization, log-transformation, PCA, kNN graph, UMAP, and Leiden clustering at resolution 0.4). The resulting embedding was dominated by slide identity and produced extreme over-fragmentation of the graph partition (1,291 Leiden clusters at resolution 0.4), indicative of strong slide/panel-driven effects and insufficient shared biological signal for robust integrated clustering (Fig. 5). These observations support our design choice to perform the refinement step slide-by-slide: within a slide, the full Xenium panel is available (maximizing gene signal), technical covariates are more homogeneous, and clusters are more stable and interpretable for marker-based differential expression.

### Generating H&E patches with corresponding segmentation and classification masks

For each slide, the Xenium SpatialData object provides (i) an H&E WSI already co-registered to the Xenium coordinate system, (ii) one nucleus polygon per segmented cell (nucleus_boundaries, keyed by cell_id), and (iii) a per-cell table containing the final ST-derived cell-type annotation. We converted the per-cell annotation into a fixed set of mask categories through a deterministic mapping

$$g:{\mathtt{final}}\_{\mathtt{label}}\to \{1,\ldots ,C\},$$

with index 0 reserved for background. For this step, *C* = 9 for the detailed label set (Epithelial, Blood_vessel, Fibroblast_Myofibroblast, Myeloid, B_Plasma, T_NK, Melanocyte, Specialized, and Other). Other category groups Unknown, Stem_like, and Less10 labels. Importantly, the nucleus geometry defined the Xenium nucleus segmentation coming from DAPI, while the nucleus class was inherited from the corresponding cell_id annotation using whole-cell RNA count matrices. To avoid incorporating excessively specialized tissues, such as those containing unique tumor cell populations or distinctive tissue architectures, the slides brain_s0, bone_marrow_s0, bone_marrow_s1, and bone_s0 were excluded at this stage.

At a given magnification level (we used 40× resolution for analysis, but 20× resolution is also provided in Data records), the H&E WSI was tiled into square patches of size *W* × *W* (here *W* = 256) on a regular grid with overlap *O* (here *O* = 64), producing patch bounding boxes {*B*_*k*_} in the intrinsic H&E pixel coordinate system. For each patch *B*_*k*_, we put the nucleus polygons into the intrinsic H&E pixel coordinate system, and we rasterize all nucleus polygons intersecting *B*_*k*_ to produce a tensor

$${{\bf{M}}}_{k}\in {{\mathbb{N}}}^{W\times W\times (C+1)}.$$

Channels 1, …, *C* store instance IDs per class, while the last channel stores the pixel-wise type index. Instance IDs are local to each patch and start at 1. This representation follows the PanNuke format. The resulting masks were subsequently converted to the CellViT format, which separates the representation into an instance map and a type map. The pseudo-code for mask generation is provided in Listing 1.

#### Listing 1

Principle of mask generation and formatting. 

Because patches overlap and nuclei can be truncated at patch borders, the same biological cell_id may appear in multiple patches. Each occurrence is treated as a distinct *patch-level instance* with its own patch-local instance ID, while the type label remains the same via *g*(*final*_*label*). The last channel of the PanNuke tensor (and the CellViT type_map) therefore represents the ST-derived cell-type category propagated to nuclear pixels through the cell_id linkage.

## Data Records

The dataset^35^ is stored in the BioStudies database^36^ and can be accessed online at 10.6019/S-BIAD2146. The cell and patch IDs are kept consistent across all data. To support reproducibility, data related to our technical validation experiments is also provided. For example, the sdata tables (features_cellvit, features_phikonv2, features_vit_google, and table_scvi), the singleR labels, the proxy metrics for label confidence, the PanNuke labels, and the CellViT fine-tuning files and results relate to content described in the technical validation section below.

### A SpatialData object for each slide in .zarr format

In the **“sdata_slides”** folder, a SpatialData object named **“sdata_{slide_id}.zarr.zip”** is provided for each slide with all the information and analyses. The SpatialData format can be manipulated using all the tools in the Scverse ecosystem^20^, making it easy and convenient to use. This SpatialData object includes the following components:

- **“he” image**: H&E slide at several levels (with level 0 corresponding to 40× resolution and level 1 corresponding to 20× resolution), aligned with ST and DAPI.
- **“morpho” image**: DAPI image at several levels showing each nucleus.
- **“st” points**: Transcript locations on the slide.
- **“cell_boundaries” shapes**: Polygons for cell boundaries with corresponding cell ID.
- **“nucleus_boundaries” shapes**: Polygons for nucleus boundaries with same corresponding cell ID.
- **“he_patches” shapes**: Coordinates for H&E patches of size 256 px with 64 px overlap at 40× resolution, with metrics (Dice coefficient, Jaccard index, and Panoptic Quality) that compare the given segmentation mask with pre-trained CellViT prediction.
- **“table_cells” table**: Table that gives the RNA counts for each cell, along with preprocessing layer, cell type annotation, cell ID, and clustering.
- **“table_nuclei” table**: Table that gives the RNA counts for each nucleus, along with preprocessing layer, cell type annotation, cell ID, clustering, and PanNuke label.
- **“table_combined” table**: Table for MFA analysis with RNA counts for cytoplasm and for nucleus, along with clustering analysis and cell type annotation.
- **“features_cellvit” table**: Table that gives the nucleus embeddings for each cell using the pre-trained CellViT model as encoder.
- **“features_phikonv2” table**: Table that gives the cell embeddings for each cell using the Phikon-v2 model as encoder.
- **“features_vit_google” table**: Table that gives the cell embeddings for each cell using the ViT-based model from Google as encoder.
- **“table_scvi” table**: Table that gives the scVI embeddings for each cell, using scVI model specifically trained for each slide.

#### *NOTE*: Contents of “table_cells” and “table_nuclei”

Both tables are stored as AnnData tables and share the same internal organization (layers, obs, obsm, obsp). They contain one row per segmented biological cell in the slide, and differ only by the RNA compartment:

- **Layers**. (layers) **“count”** stores the raw integer gene-count matrix; **“log_norm”** stores the library-size-normalized and log-transformed expression used for PCA, kNN graph construction, UMAP, and Leiden clustering.
- **Cell-level metadata**. (obs) **“cell_id”** (unique identifier within the slide); **“final_label”** (harmonized cell-type category used throughout STHELAR, categories are Epithelial, Blood_vessel, Fibroblast_Myofibroblast, Myeloid, B_Plasma, T_NK, Melanocyte, Glioblastoma, Specialized, Stem_like, Unknown, and Less10.); **“label1”,** **“label2”,** **“label3”** (intermediate, slide-specific curation levels); **“transcript_counts”** (total detected transcripts); graph-clustering labels of the form **“pca_n{N}_pcs{N_pcs}_leiden_res{res_value}”** (Leiden cluster IDs computed after PCA on log_norm, typically for res_value ∈ {0.2, 0.4, 0.6}); histology-derived unsupervised cluster IDs **“kmeans_clustersHE_cellvit”,** **“kmeans_clustersHE_phikonv2”**, and **“kmeans_clustersHE_vit_google”** (k-means on H&E encoder embeddings); and CellViT/PanNuke-derived nucleus phenotype fields **“PanNuke_label”** and **“PanNuke_proba”**. In addition, table_nuclei contains **“ct_tangram”** (fine-grained Tangram-predicted cell-type label from nucleus RNA counts).
- **Multi-dimensional embeddings**. (obsm) **“spatial”** stores the 2D coordinates of each cell in the slide reference frame; **“X_pca”** stores PCA coordinates computed from log_norm; **“X_umap”** stores the corresponding UMAP embedding.
- **Cell-cell graphs**. (obsp) **“pca_n{N}_pcs{N_pcs}_connectivities”** and **“pca_n{N}_pcs{N_pcs}_distances”** store the weighted kNN graph (connectivities) and neighbor distances used for Leiden clustering and UMAP, matched to the PCA parameterization indicated in the key.

Having the precomputed X_umap embedding available in obsm is particularly valuable because, for slides with a huge amount of cells, computing UMAP (and its underlying kNN graph) can be computationally demanding and may require GPU-accelerated toolkits such as rapids-singlecell. Therefore, providing these embeddings enables users to immediately visualize and explore slide-level structure and annotations without having to rerun heavy dimensionality-reduction workflows.

### A table summarizing all information related to cell-type labels

To enable users to easily access all information related to cell type labels, a summary table is provided for each slide ID. This table compiles, for all cell IDs, the nucleus and whole-cell cell type labels, alternative labels, as well as the associated confidence proxy metrics. The table is available for each slide ID at **“summary_all_labels_per_slide/{slide_id}_cell_metadata.parquet.”**. It contains the following columns:

- **“cell_id”**: Unique identifier of the biological cell within the given slide.
- **“nuclei_ct_tangram”**: Fine-grained cell-type label predicted by Tangram using nucleus RNA counts.
- **“nuclei_ct_tangram_group”**: Grouped version of nuclei_ct_tangram, obtained by mapping Tangram’s fine labels to the harmonized label space used in STHELAR.
- **“nuclei_label1”**: Curated nucleus-derived label assigned after Tangram-guided and marker-gene-driven refinement, level 1 annotation for nuclei (first-stage label used during refinement; slide-specific granularity).
- **“nuclei_label2”**: Curated nucleus-derived label assigned after Tangram-guided and marker-gene-driven refinement, level 2 annotation for nuclei (more precise/secondary label level; slide-specific granularity).
- **“nuclei_label3”**: Attempt to label cancer versus normal cell, level 3 annotation for nuclei.
- **“nuclei_final_label”**: nuclei_label1 harmonized across slides. Categories are Epithelial, Blood_vessel, Fibroblast_Myofibroblast, Myeloid, B_Plasma, T_NK, Melanocyte, Glioblastoma, Specialized, Stem_like, Unknown, and Less10.
- **“nuclei_final_label_group”**: Grouped version of nuclei_final_label, where low-information categories are collapsed (Less10, Unknown, Stem_like to Other).
- **“cells_label1”**: Curated whole-cell-derived label assigned after Tangram-guided and marker-gene-driven refinement, level 1 annotation for cells (first-stage label used during refinement; slide-specific granularity).
- **“cells_label2”**: Curated whole-cell-derived label assigned after Tangram-guided and marker-gene-driven refinement, level 2 annotation for cells (more precise/secondary label level; slide-specific granularity).
- **“cells_label3”**: Attempt to label cancer versus normal cell, level 3 annotation for cells.
- **“cells_final_label”**: cells_label1 harmonized across slides. Categories are Epithelial, Blood_vessel, Fibroblast_Myofibroblast, Myeloid, B_Plasma, T_NK, Melanocyte, Glioblastoma, Specialized, Stem_like, Unknown, and Less10. **These are the labels used in the various analyses**.
- **“cells_final_label_group”**: Grouped version of cells_final_label, where low-information categories are collapsed (Less10, Unknown, Stem_like to Other). **These are the labels used to construct the patches for CellViT fine-tuning below**.
- **“cells_transcript_counts”**: Total number of detected transcripts assigned to the whole cell (library size for that cell; used both for QC and as an RNA-depth metric proxy).
- **“cells_singleR”**: Fine-grained reference-based label predicted by SingleR from whole-cell RNA counts.
- **“cells_singleR_group”**: Grouped version of cells_singleR, obtained by mapping SingleR’s fine labels to the harmonized label space used in STHELAR.
- **“cells_final_label_confidence”**: Neighborhood purity score  = 1 − normalized Shannon entropy of the full kNN label distribution induced by connectivity weights ([0,1]; higher = more locally unambiguous). Also named ct_confidence in the analysis later.
- **“cells_final_label_prob”**: Assigned-label agreement  = *p*_*i*_ (assigned label), i.e. the connectivity-weighted fraction of neighbor mass matching the cell’s label. Also named ct_support in the analysis later.
- **“cells_final_label_alt1”**: First alternative label according to the kNN-neighborhood label distribution.
- **“cells_final_label_alt1_prob”**: Probability/support associated with cells_final_label_alt1 in the kNN-neighborhood label distribution.
- **“cells_final_label_alt2”**: Second alternative label according to the kNN-neighborhood label distribution.
- **“cells_final_label_alt2_prob”**: Probability/support associated with cells_final_label_alt2.
- **“cells_final_label_alt3”**: Third alternative label according to the kNN-neighborhood label distribution.
- **“cells_final_label_alt3_prob”**: Probability/support associated with cells_final_label_alt3.
- **“cells_knn_row_sum”**: Total kNN-graph connectivity weight for the cell (row-sum of the Scanpy connectivities matrix, equals the cell’s weighted degree/strength in the neighbor graph, after self-edge removal). Low values indicate weak/absent neighborhood connectivity and can make confidence/support undefined (NaN when row-sum is 0).
- **“cells_rna_depth_log1p”**: Log-transformed RNA depth proxy computed as $\log (1+c)$ where *c* = *cells*_*transcript*_*counts*, providing a stabilized per-cell library-size measure.
- **“cells_rna_depth_quantile”**: Within-slide rank-based quantile of RNA depth ( ∈ [0, 1]), enabling comparison of RNA abundance across cells in the same slide independently of panel size and scale.
- **“combined_final_label”**: Label obtained from the table_combined (integration of nuclear and cytoplasmic RNA information using MFA analysis).

***NOTE:*** ct_tangram, label_1, label_2, label_3, final_label, and transcript_counts are the same information provided previously in the table_cells and table_nuclei in *1. A SpatialData object for each slide in .zarr format*.

***NOTE:*** ct_tangram, label_1, label_2, and label_3 are intermediate labels used during the annotation process. Users should rely on final_label for downstream analyses and interpretation. In particular, cells_final_label_group contains the labels used for segmentation and classification masks below.

***NOTE:*** The per-cell confidence scores reported above should be interpreted as proxy uncertainty metrics rather than true probabilities of being correctly annotated.

### H&E patches with corresponding segmentation and classification masks

In the **“data_40x/data”** folder, we provide:

- **“images.zip”**: H&E patches for all the slides together, using {slide_id}_{patch_num} identification.
- **“masks_slides”** folder: Contains a **“masks_{slide_id}.npz”** file for each slide containing segmentation and cell type classification masks, formatted identically to those in PanNuke.
- **“annot_dicts_ct_1”** folder: Contains the **“label2cat.json”** dictionary which maps the final cell labels in sdata to categories in the classification masks, the **“cat2color.json”** dictionary which maps each category in the masks to a plotting color for visual inspection, and the **“cat2idx.json”** dictionary which maps each category to its corresponding index position within the mask.
- **“masks_cell_ids_nuclei.zip”**: Segmentation masks for all the slides together, assigning to each pixel the cell ID corresponding to its nucleus.
- **“pretrained_CellViT_mask_preds”** folder: Contains a **“instance_map_predictions_{slide_id}.h5”** file for each slide with the predicted segmentation for each patch using the pre-trained CellViT model.

In this folder, we also provide the **“patches_overview_sthelar40x.parquet”** file. This is a global patch-level summary file for all slides together. It enables rapid dataset-level exploration and filtering without loading the images or per-cell metadata, and provides access to each patch’s coordinates on its originating slide. This Parquet table contains one row per H&E patch and includes the following columns:

* slide_id: Identifier of the originating WSI.

* file_name: {slide_id}_{patch_num} identification.

* xmin, ymin, xmax, ymax: Patch coordinates in slide space.

* Dice, Jaccard, bPQ: Segmentation quality metrics.

* T_NK, B_Plasma, Myeloid, Blood_vessel, Fibroblast_Myofibroblast, Epithelial, Specialized, Melanocyte, Other: Number of cells present in the patch for each cell-type label.

### Data for CellViT fine-tuning

For the global dataset, the following element can be found in the **“data_40x/data”** folder:

- **“cell_count.csv”**: Table summarizing, for each patch ID across all slides, the nucleus count per cell type. It follows the same format as the cell_count.csv file provided by the CellViT authors. Counts are *patch-level cell instances* (cells are counted once per patch in which they appear), and therefore exceed the number of unique biological cells.
- **“labels.zip”**: Segmentation and classification masks for all slides. Unlike the previous masks_{slide_id}.npz files, this format follows the CellViT convention, which separates the original mask into an instance mask and a cell type mask.
- **“patch_metrics.csv”**: Table storing the evaluation metrics for each mask, by comparing with the predicted mask from the pre-trained CellViT model. Metrics are Dice coefficient, Jaccard index, and Panoptic Quality. These metrics enable user-defined quality filtering.
- **“types.csv”**: Table giving the tissue type for each patch.

Two fine-tunings have been done with detailed or grouped cell type classes. We provide for each fine-tuning in **“data_40x/finetuning_CellViT_detailed”** or **“data_40x/finetuning_CellViT_grouped”** folders:

- **“cell_count_train.csv”,** **“cell_count_valid.csv”**, and **“cell_count_test.csv”**: Split versions of the cell_count.csv file indicating which patch belongs to the train, validation, and test sets. Splits are performed within each slide (i.e., same WSIs across subsets).
- **“dataset_config.yaml”** and **“weight_config.yaml”**: Configuration files required for fine-tuning CellViT.

### CellViT fine-tuning outputs

In each fine-tuning directory (**“data_40x/finetuning_CellViT_detailed”** or **“data_40x/finetuning_CellViT_grouped”**), the subfolder **“output”** contains all results from the fine-tuning and evaluation processes:

- **“config.yaml”**: Configuration file used for CellViT fine-tuning.
- **“logs.log.1–5”**: Log files for fine-tuning.
- **“checkpoint_n.pth”**: Checkpoint for the best model with n the corresponding epoch number.
- **“inference_results.json”**: File containing inference results on the test set.
- **“inference.log”**: Log file for inference.

### Analysis of the fine-tuned CellViT models

For each fine-tuning, the resulting model was applied to all slides. Results can be found in the **“data_40x/finetuning_CellViT_detailed/finetuned_model_analysis”** or **“data_40x/finetuning_CellViT_grouped/finetuned_model_analysis”** folders, which contain a subfolder for each slide ID with the following files:

- **“config.yaml”**: Configuration from the fine-tuning.
- **“inference_results.json”**: Prediction results.
- **“inference.log”**: Log file for inference.
- **“inference_instance_map_predictions.h5”**: Predicted segmentation mask for each patch for all the slides.
- **“cell_features_cellvit.npy”**: Nucleus embeddings for each cell using the given fine-tuned CellViT model as encoder.
- **“pixel_class_gt_mask.pth”**: The pixel count for each cell type category for each nucleus using Xenium-based segmentation mask instead of the predicted one.

All analyses were conducted on H&E patches at 40× resolution. However, images.zip, labels.zip, masks_cell_ids_nuclei.zip, instance_map_predictions_all_slides.h5 (segmentation prediction using pre-trained CellViT), patches_overview_sthelar20x.parquet, patch_metrics.csv, cell_count.csv, and types.csv are also provided at 20x resolution in **“data_20x/data”** folder for research purposes.

To facilitate access to this dataset for model training tasks, we extracted a subset containing only the H&E patch images, along with their cell ID masks, and uploaded it to HuggingFace in parquet format, with corresponding cell type summary tables. The 40× resolution is available at 10.57967/hf/6008^37^, and the 20× resolution at 10.57967/hf/6009^38^. Each entry includes the following information:

- **file_name**: Filename of the H&E image patch (e.g., breast_s0_10.png).
- **slide_id**: Identifier of the slide from which the patch was extracted (e.g., breast_s0).
- **tissue**: Tissue type, provided as categorical labels (e.g., Breast, Lung, Colon).
- **image**: RGB color images of size 256 × 256 px, extracted from H&E-stained whole-slide images at 40× or 20× magnification, with a 64 px overlap between adjacent patches.
- **cell_id_map**: Sparse segmentation mask (CSR matrix stored as .npz bytes) aligned with the H&E patch, where each nucleus pixel stores its cell ID integer (0 = background). This is the cell-identity mask, not the ’instance index’ mask: the integer values are stable per slide and are designed to join with the per-slide metadata parquets mentioned below.
- **Dice**: Dice similarity coefficient comparing the provided segmentation mask with the segmentation mask predicted by the pre-trained CellViT model (SAM-H encoder).
- **Jaccard**: Jaccard index comparing the provided segmentation mask with the segmentation mask predicted by the pre-trained CellViT model (SAM-H encoder).
- **bPQ**: Binary Panoptic Quality score comparing the provided segmentation mask with the segmentation mask predicted by the pre-trained CellViT model (SAM-H encoder).

The **“summary_all_labels_per_slide/{slide_id}_cell_metadata.parquet”** files are also provided, and the associated cell_id_map masks enable direct correspondence between the cell_id entries in the table and the nuclei in the segmentation masks.

Providing the cell_id_map in HuggingFace is more flexible than providing the instance_map and type_map. This allows the user to trace back to the initial data and to the various analyses stored in BioStudies for each cell ID and each slide ID, and to adjust the labels according to their convenience. We also provide the **patches_overview_sthelar{res}x.parquet** file to facilitate dataset exploration and filtering, and access to patch coordinates.

## Data Overview

**Fig. 6:**
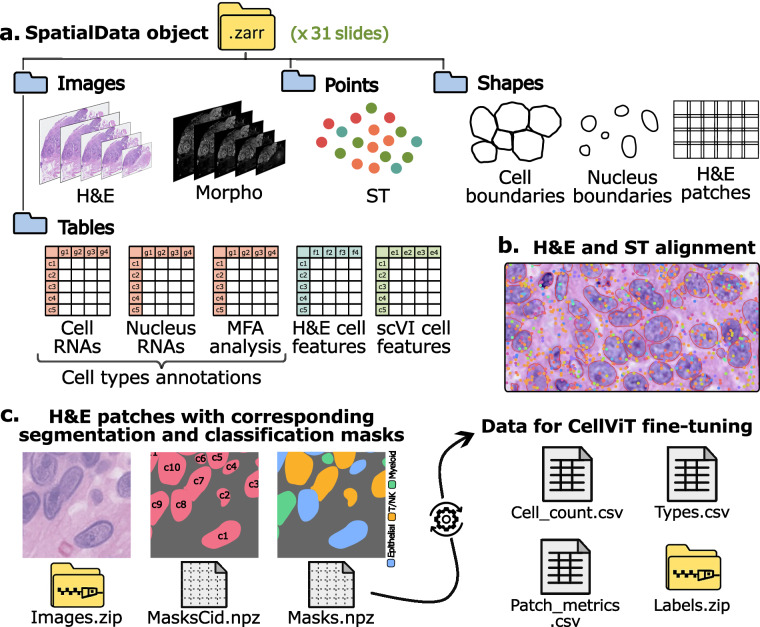
Summary of the dataset. **a**. For each slide, a SpatialData object is provided, including: **H&E**: the H&E-stained WSI at multiple resolutions, **Morpho**: DAPI-stained images for nuclear morphology at multiple levels, **ST**: spatial transcriptomics data showing RNA molecule positions, **Cell/Nucleus boundaries**: cell and nucleus segmentation masks, **H&E patches**: coordinates for 40×H&E patches of 256 × 256 px with 64 px overlap, **Cell/Nucleus RNAs**: RNA count tables per cell and nucleus, along with cell type annotations and clustering labels (table_cells and table_nuclei), **MFA analysis**: results of a multi-factor analysis (MFA) integrating cytoplasmic and nuclear RNA counts (table_combined), **H&E cell features**: extracted H&E features from multiple encoder models (features_cellvit, features_phikonv2, and features_vit_google), and **scVI cell features**: cell-level embeddings from scVI (table_scvi). **b**. Example illustrating the alignment between the H&E and ST modalities. **c**. Additional files include: **Images.zip**: a ZIP archive containing all H&E patches at 40× resolution (images.zip), **Masks.npz**: per-slide files with cell type masks matching the PanNuke format (masks_{slide_id}.npz), and **MasksCid.npz**: cell ID segmentation masks (masks_cell_ids_nuclei.zip). Data formatted for use with CellViT are also provided, including: **Cell_count.csv**: nucleus count per cell type per patch ID, **Types.csv**: tissue type for each patch, **Patch_metrics.csv**: comparison between Xenium-based segmentation mask and pre-trained CellViT segmentation prediction, and **Labels.zip**: segmentation and classification masks for all the slides matching the input CellViT format. For model fine-tuning section, 20× resolution images and their corresponding masks and metrics are also included.

**Fig. 7:**
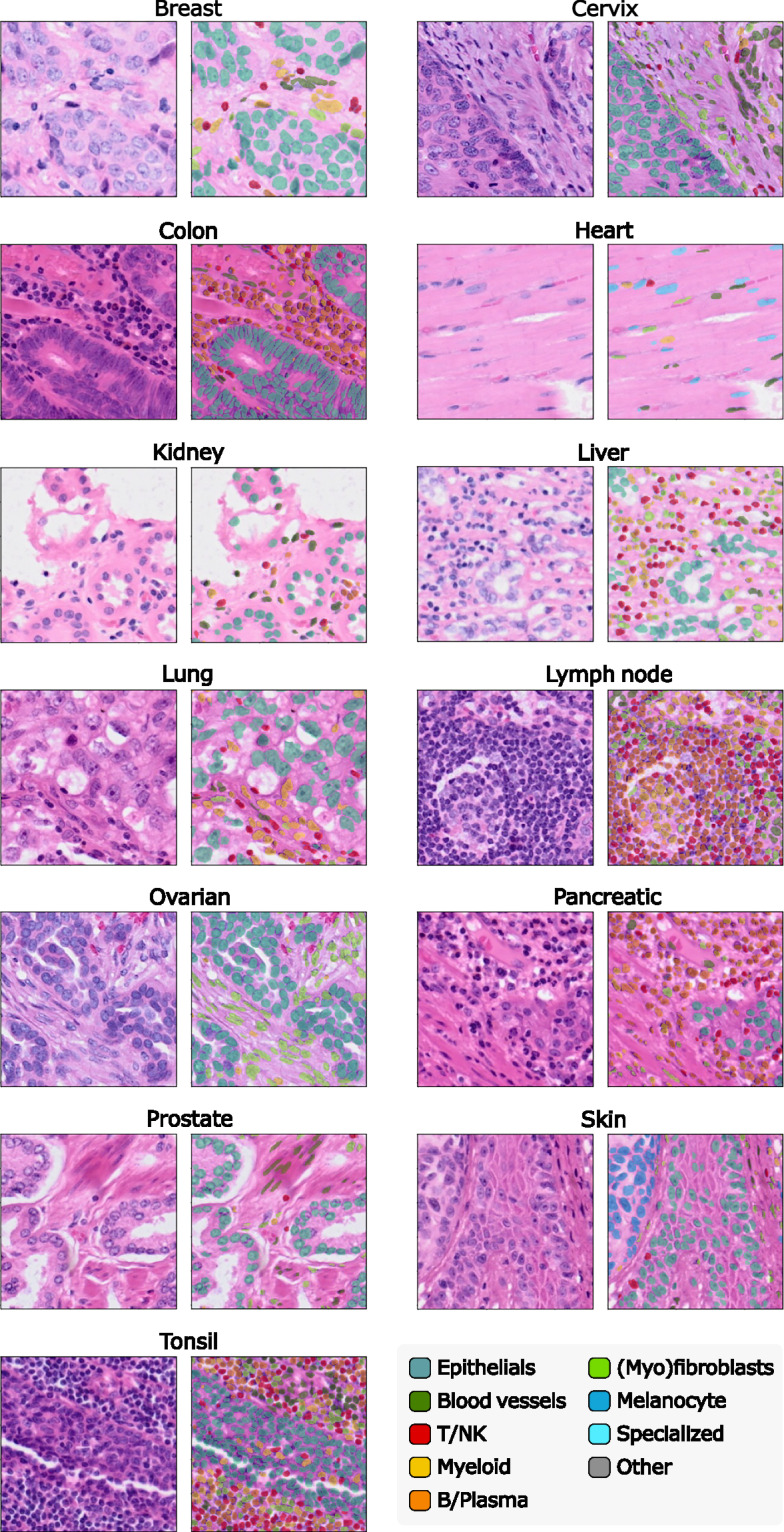
Example of 20× resolution H&E patches with corresponding segmentation and classification masks for each tissue type.

This work presents a large-scale cell-type annotated dataset derived from 10× Genomics Xenium slides. It includes 31 WSIs with 16 tissue types, each processed into a comprehensive multi-modal object. For each slide, we provide per-cell and per-nucleus transcript count tables with clustering results and standardized cell type annotations, scVI-derived embeddings, cell feature vectors from multiple H&E encoders, nuclei and cell segmentation boundaries, co-registered DAPI and H&E images, and patch coordinates with segmentation metrics. These components are included in a single SpatialData object per slide, with unique identifiers per cell and nucleus to facilitate integration, visualization, and downstream analysis using the Scverse ecosystem^20^. In total, the dataset contains over 11 million *unique biological cells* before patch extraction. The dataset also includes 587,555 H&E image patches at 40× and 154,814 image patches at 20×, with segmentation and cell type classification masks. When counting cell annotations at the patch level, the total number of annotated nuclei instances increases to  ~19.7M due to overlap-based duplication. This value should be interpreted as the size of the patch-instance training corpus, not as the number of distinct cells. A visual summary of the resource is provided in Fig. 6. For each tissue type, an example patch at 20×resolution along with its corresponding classification mask is shown in Fig. 7.

## Technical Validation

### Cell type annotation - Quantitative assessment of Tangram–Final label concordance

**Fig. 8:**
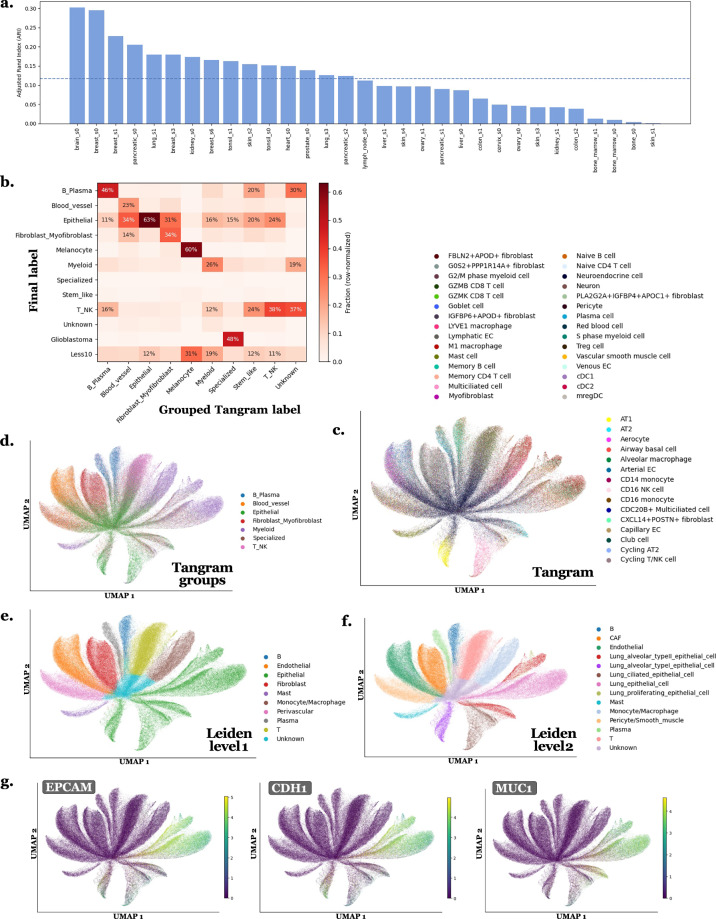
Quantitative and qualitative assessment of annotation stability between Tangram and final labels. This is using *nucleus RNA count matrices*. The “Tangram groups” correspond to the Tangram labels manually grouped into the corresponding final label categories. **a**. Adjusted Rand Index (ARI) computed between grouped Tangram labels and final standardized labels for each slide, with the dashed line indicating the mean ARI across all slides. **b**. Row-normalized confusion matrix aggregated across all slides between grouped Tangram labels (columns) and final labels (rows). **c**. UMAP of slide lung_s3 colored by original fine-grained Tangram labels (“Tangram”). **d**. Same UMAP colored by grouped Tangram labels mapped to the final label categories (“Tangram groups”). **e**. Same UMAP colored by intermediate high-level manual labels after Tangram and marker-gene integration (“Leiden level 1”) before cross-slide standardization. **f**. Same UMAP colored by intermediate low-level manual labels after Tangram and marker-gene integration (“Leiden level 2”) before cross-slide standardization. **g**. Example of marker-gene expression for a cluster exhibiting Tangram–marker discordance.

To quantitatively assess the stability and consistency of the final labels with respect to Tangram-derived annotations, we compared the final standardized labels with Tangram predictions mapped to the same final label space, using nucleus RNA count matrices. For this purpose, the Adjusted Rand Index (ARI) was computed between grouped Tangram labels and final labels independently for each slide (Fig. 8a). In addition, a global confusion matrix aggregated across all slides was computed between grouped Tangram labels and final labels, and visualized in row-normalized form (Fig. 8b). This analysis reveals a moderate concordance, consistent with Tangram being used as a guiding signal rather than a strict labeling rule. To illustrate how conflicts were resolved in practice, Fig. 8c–g shows an example for slide lung_s3. For instance, two clusters initially suggested as myeloid by Tangram were reassigned as epithelial based on the expression of epithelial markers such as *EPCAM*, *CDH1*, and *MUC1* (Fig. 8g). This example illustrates the decision rule used throughout the dataset, where Tangram provides an informed prior, but final annotation is driven by marker-gene evidence to ensure biological plausibility and internal transcriptional consistency rather than strict adherence to the reference atlas.

### Cell type annotation - Impact of boundary selection on cell type annotations

While nucleus segmentation comes from DAPI staining for all the slides, cell segmentation from 10× Genomics is determined by two main methods according to available data. The first nuclear expansion method approximates cell boundaries by isotropically expanding the nuclear boundaries until they encounter adjacent cells or a predefined limit. The second method, a multimodal approach that uses additional cytoplasmic and membrane staining in conjunction with DAPI staining, yields more accurate boundaries^16^. The cell segmentation modality used for each slide is reported in Table 3. Cell boundaries critically determine the RNA count attributed to each gene within individual cells, and thus inaccuracies in cell boundary delineation directly influence RNA quantification, potentially resulting in inaccurate cell annotations. Conversely, only relying on more precise nucleus boundaries risks omitting significant cytoplasmic gene expression information. To evaluate the impact of boundary selection on cell type annotations, additional analyses were performed. As previously detailed, the annotation process was conducted twice: first using the *nucleus RNA count matrices* and then using the *whole-cell RNA count matrices*. Additionally, we employed another annotation approach by applying Multiple Factor Analysis (MFA)^39^ to the cytoplasmic and nuclear RNA count data, followed by Leiden clustering again. Within each resulting cluster, we compared the proportions of annotations derived exclusively from nuclear data to those derived exclusively from whole-cell data. Clusters showing high agreement adopted labels from the whole-cell approach, while clusters with significant discrepancies underwent additional differential expression analysis to assign definitive labels. Then, the concordance between these three labels (i.e., “cells“, “nuclei“, and “MFA” labels) was analyzed. The concordance was defined here as the percentage of exact per-cell_id label matches between two given annotation strategies. Formally, for two label assignments *A* and *B* defined over the same set of cells, concordance was computed as

$$\,{\rm{Concordance}}\,(A,B)=100\times \frac{1}{N}\mathop{\sum }\limits_{i=1}^{N}{\bf{1}}[{A}_{i}={B}_{i}],$$

where *N* is the number of cell IDs included in the comparison. This definition corresponds to a strict, per-cell identity check. Concordance was computed independently for each slide and also across the pooled dataset of all slides. Concordance for four complementary comparisons was evaluated: (i) “cells” versus “nuclei” labels using all the cell IDs, (ii) “cells” versus “nuclei” after exclusion of cell IDs with low-RNA, (iii) “cells” versus “MFA” for all the cell IDs, and (iv) “cells” versus “MFA” after exclusion of cell IDs with low-RNA. Low-RNA cells were defined as cells containing fewer than 10 detected transcripts and labeled as Less10.

**Fig. 9:**
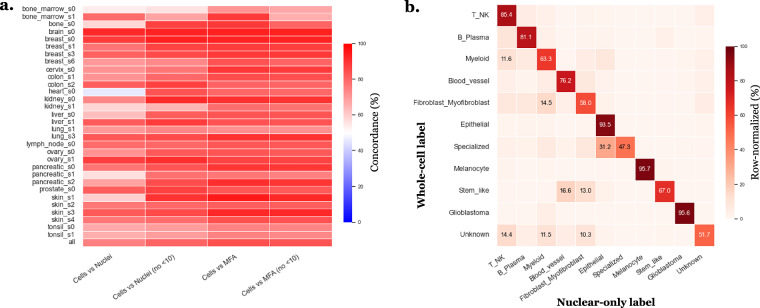
Comparison between nuclear-only and whole-cell annotations. **a**. Heatmap showing per-slide concordance values computed as the percentage of exact per-cell label matches between different annotation strategies: “cells” versus “nuclei” labels, and “cells” versus “MFA” labels. For each comparison, concordance is reported both with and without exclusion of low-RNA cells (cell IDs labeled Less10, i.e., containing fewer than 10 detected transcripts). Each row corresponds to one slide, and the bottom row (“all”) reports concordance computed on the pooled dataset across all slides. **b**. Row-normalized confusion matrix (aggregated across all slides) comparing “cells” labels (rows) to “nuclei” labels (columns), after excluding cells with fewer than 10 transcripts (Less10). Each entry represents the percentage of cells of a given whole-cell type assigned to each nuclear-only type.

Figure 9a reports the resulting concordance values for all comparisons. For the “Cells vs Nuclei (no  <10)” comparison, the overall concordance was 80.4% ± 8.9, and for the “Cells vs MFA (no  <10)” comparison, the overall concordance was 83.3% ± 7.4. To further characterize the nature of annotation differences beyond a single summary score, we also computed a row-normalized global confusion matrix between “cells” and “nuclei” labels, aggregated across all slides after exclusion of cells with fewer than 10 RNAs (Fig. 9b). Row-wise agreement ranges from 47.3% (Specialized) to 95.7% (Melanocyte), with high concordance for Epithelial ((93.5%)), Glioblastoma ((95.6%)), T_NK ((85.4%)), B_Plasma ((81.1%)), and Blood_vessel ((76.2%)). The remaining differences were concentrated in a few classes, notably Specialized being reassigned to Epithelial in the nucleus-based labels ((31.2%)) and Fibroblast_Myofibroblast showing cross-assignment with Myeloid ((14.5%)). Disagreements are also driven by Unknown and Stem_like cells, consistent with their limited marker-gene support. As noted previously, the “cells” label was selected for the final construction of the dataset.

### Cell type annotation - scVI validation

**Fig. 10:**
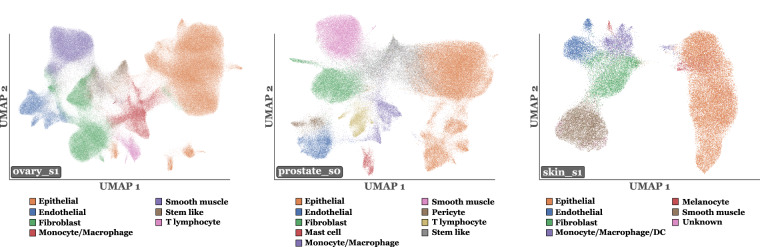
UMAPs from scVI embeddings for three example slides ovary_s1, prostate_s0 and skin_s1. A scVI model was trained on each slide separately using the RNA count tables for the cells. UMAPs were then performed on the embeddings obtained with the trained scVI model. Labels from the annotation process with Tangram + Leiden clustering are used.

Further validation of cell annotations was achieved using the single-cell Variational Inference (scVI) model, a variational autoencoder (VAE)-based deep learning framework specifically designed for single-cell RNA sequencing analysis^24^. scVI models were trained independently on each slide. UMAP visualizations of scVI embeddings, colored by our final cell labels, facilitated the qualitative assessment of annotation consistency. Results generally demonstrate strong agreement, with illustrative examples shown in Fig. 10.

### Cell type annotation - SingleR-based external validation

Because cell-type annotation is a critical step that will subsequently serve as the ground truth, ensuring maximal reliability is essential. A recent benchmarking study from Cheng, J., Jin, X., Smyth, G.K. *et al*.^40^ aimed to evaluate the performance of several computational methods for assigning cell types in imaging-based spatial transcriptomics data, specifically using the 10× Xenium platform. The authors compared SingleR, Azimuth, RCTD, scPred, and scmapCell methods, and concluded that SingleR achieved the best overall performance. It is therefore of interest to compare the annotations produced by SingleR with our final cell-type labels. An important point to note, however, is that the benchmarking study uses manual annotation based on known marker genes as ground truth, which is precisely the strategy we adopted to refine our final cell-type labels. It suggests that, despite its lack of scalability, marker gene–based manual annotation remains the most reliable approach, offering the highest level of confidence. This further highlights the value of our dataset, in which each cluster was carefully examined manually using established marker genes. In our case, scalability is not the primary objective, and the main contribution lies in producing a highly reliable reference dataset. Nevertheless, it remains informative to compare our annotations with those obtained using SingleR.

**Fig. 11:**
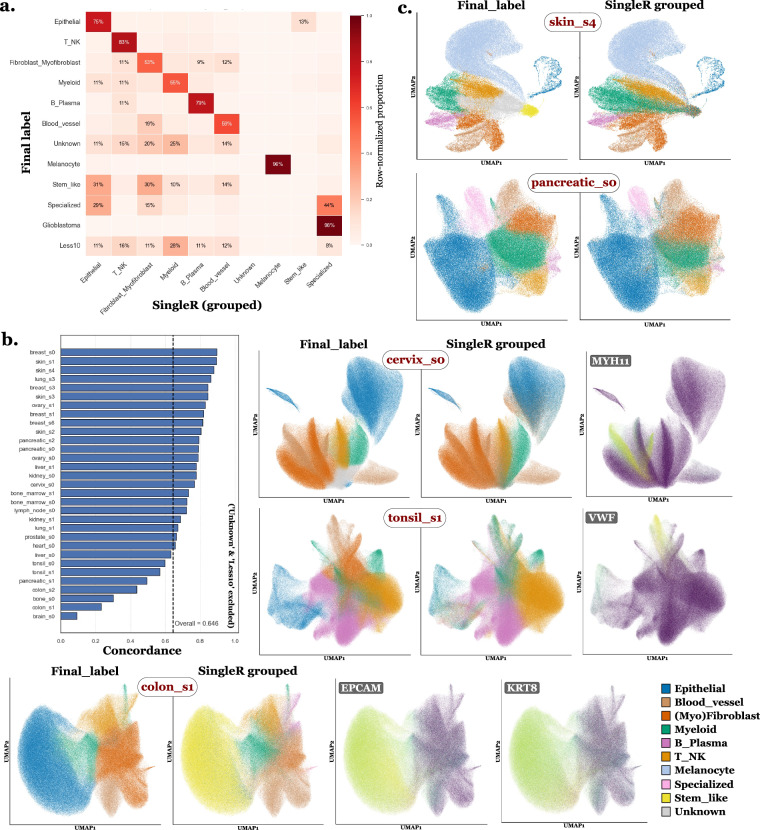
Independent validation of Xenium cell-type labels using SingleR on whole-cell RNA count matrices. **a**. Global confusion matrix comparing the final cell-type labels to reference-based SingleR grouped annotations computed from the same whole-cell RNA count matrices. Fine-grained SingleR reference labels were manually mapped to the coarser cell-type final categories, using the same mapping dictionary used for Tangram comparison. Values are row-normalised proportions. **b**. Concordance per slide, defined as the fraction of cells with identical final label and grouped SingleR assignments (excluding Less10 and Unknown from the accuracy calculation). The dashed vertical line indicates the global concordance across slides. **c**. UMAP examples for some slides showing RNA-based cell embeddings coloured by final label (left) and grouped SingleR (right). For slides with mismatches (cervix_s0, tonsil_s1, colon_s1), additional marker-gene overlays were inspected for discordant clusters.

Accordingly, we additionally performed reference-based cell-type assignment using SingleR on *whole-cell RNA count matrices*. For each slide, SingleR was run using the same corresponding reference atlases as those used for Tangram (Table 5), after restricting the analysis to the intersection of genes between the query and reference datasets and using log-normalized counts. The SingleR outputs were then mapped to the final annotation categories using the same mapping dictionary as applied for Tangram. Figure 11 summarizes the comparison between the final cell-type labels and the labels assigned by SingleR. While a good overall correspondence between the two annotation schemes is observed, several conflicts remain. UMAP visualizations for selected slides illustrate that, although cluster-level agreement is generally strong, discrepancies occur for specific clusters. Examples of marker gene expression indicate that marker-based manual annotation appears more reliable for defining the final cell type in these cases. This is consistent with the benchmarking study’s use of manual annotation as ground truth and further supports the relevance of our approach as a final annotation strategy.

It is essential to note that we applied SingleR as an a posteriori external validation rather than as the primary labeling strategy. The dataset labels were historically derived from the Tangram prior, followed by the slide-specific clustering and manual marker-gene review. The agreement between SingleR and the curated labels supports the annotation method, while preserving SingleR as an independent validation axis.

### Cell type annotation - Per-cell uncertainty

To address label uncertainty beyond a single definitive cell-type assignment, two complementary principles were used to assign a confidence score. The first is based on the consistency of neighboring labels within the clustering used to define the final labels, while the second relies on RNA abundance metrics.

For the first confidence metric, we retrieved the cell-cell weighted connectivity matrix stored in adata.obsp from the same kNN graph used for Leiden clustering and UMAP visualization. To prevent a cell from trivially reinforcing its own label, the diagonal of the connectivity matrix was set to zero before computation. Let *y*_*i*_ ∈ {1, …, *L*} denote the resulting final label of cell *i* (with *L* = 11 categories). For each slide, for each cell *i*, we then computed the neighborhood label distribution

$${p}_{i\ell }\ =\ \frac{\sum _{j}{w}_{ij}\,{\bf{1}}\left[{y}_{j}=\ell \right]}{\sum _{j}{w}_{ij}},$$

where *w*_*ij*_ is the kNN-connectivity weight between cells *i* and *j*. This yields a soft, graph-smoothed estimate of how consistently the local transcriptomic manifold supports each label around cell *i*. We quantified label ambiguity using Shannon entropy,

$${H}_{i}\ =\ -\mathop{\sum }\limits_{\ell =1}^{L}{p}_{i\ell }\,\log ({p}_{i\ell }+\varepsilon ),\qquad {\widetilde{H}}_{i}\ =\ \frac{{H}_{i}}{\log (L)},$$

with *ε* = 10^−12^ for numerical stability. We finally define a bounded confidence score as

$${{\rm{confidence}}}_{i}\ =\ 1-{\widetilde{H}}_{i}\in [0,1],$$

where values close to 1 indicate a locally homogeneous neighborhood (high agreement with a single label) and values close to 0 indicate a highly mixed neighborhood (high uncertainty). This score was named ct_confidence, and it answers the question “How pure/unambiguous is my neighborhood’s label mixture overall?”. In addition, we report a label-specific support score for the assigned label,

$${{\rm{support}}}_{i}\ =\ {p}_{i,{y}_{i}},$$

This score was named ct_support or prob, and it answers the question “How much do my neighbors (weighted) agree with my current label?”. We also kept the three most plausible alternative labels for each cell, defined as the top-3 labels *ℓ* ≠ *y*_*i*_ ranked by *p*_*iℓ*_, together with their associated probabilities. Moreover, we stored the neighborhood strength ∑_*j*_*w*_*ij*_ (knn_row_sum) to facilitate filtering of cells with weak graph support.

**Fig. 12:**
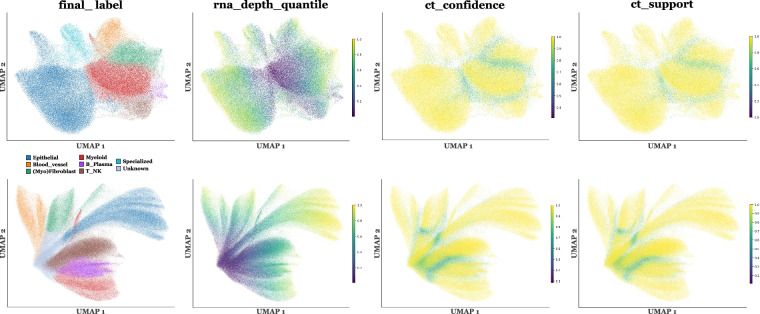
Visualization of cell type confidence proxy metrics for two slide examples. Top row: pancreatic_s0. Bottom row: breast_s6. Within each row, the UMAP is colored (left to right) by: final_label, rna_depth_quantile, ct_confidence, and ct_support.

The second alternative confidence metric is a per-cell RNA depth score. This score is based on the assumption that cells with higher RNA content within a given slide provide more reliable information for clustering and marker gene detection, thereby increasing confidence in the assignment of the final cell-type label. For each slide, we compute rna_depth_log1p$\,=\,\log (1+{\mathtt{transcript}}\_{\mathtt{counts}})$ and convert it to a within-slide quantile score rna_depth_quantile ∈ [0, 1] using rank-based normalization. This provides a robust, panel-agnostic proxy for per-cell RNA depth that is directly comparable within a slide. Figure 12 shows the results for each confidence score for two example slides displayed on UMAP embeddings.

All quantities above are released as a per-cell table for each slide, keyed by (slide_id, cell_id) and including: the original label1/2/3, the final_label, neighborhood entropy (ct_entropy, ct_entropy_norm), confidence (ct_confidence), assigned-label support (ct_support), top-3 alternative labels with probabilities (ct_alt1-3, ct_alt1_prob-ct_alt3_prob), neighborhood strength (knn_row_sum), and RNA depth metrics (rna_total_counts, rna_depth_log1p, rna_depth_quantile). This resource enables users to (i) filter cells by confidence or RNA depth, (ii) propagate uncertainty into downstream analyses, and (iii) explicitly consider alternative cell-type hypotheses in ambiguous transcriptomic regions.

The per-cell scores reported above should be interpreted as proxy uncertainty metrics rather than true probabilities of being correctly annotated. By construction, the graph-based ct_confidence/ct_support quantify local self-consistency of labels in the kNN manifold induced by the same representation used for Leiden clustering. They therefore capture ambiguity at transcriptomic boundaries and potential label mixing, but they do not validate biological correctness against an external ground truth. In particular, these scores can be inflated in globally misannotated regions that remain internally coherent, and they may be deflated for rare or transitional states, low-quality cells, or cell types that are not well separated given the targeted Xenium gene panels. Similarly, RNA-depth metrics (rna_depth_quantile) summarize within-slide relative library size and therefore mainly capture local assay yield and segmentation/capture efficiency in a rank-based way. Low RNA depth is typically associated with increased sampling noise and reduced marker detectability, but the converse does not hold: high depth does not guarantee a correct label, as abundant counts may arise from technical effects (e.g., boundary leakage, local transcript crowding, or differences in cell size) as well as from true biology. Importantly, because rna_depth_quantile is defined per slide, it is not intended for absolute comparison across slides or panels, and it should be interpreted as a contextual reliability cue rather than as a calibrated measure of annotation accuracy. Despite these limitations, releasing these quantities is valuable in practice: they enable systematic filtering, sensitivity analyses, and uncertainty-aware downstream modeling, which is preferable to treating all cell assignments as equally reliable.

### Cell type annotation - Additional validations

Additionally, to ensure that clustering was not driven by differences in total RNA content, we binned per-cell total RNA counts into seven categories and overlaid them on the UMAP for each slide (visualization not shown). The bins were well mixed across clusters, confirming that cell clustering patterns were driven by specific gene expression profiles rather than by variations in total RNA quantity.

Finally, labels were qualitatively and globally reviewed by a pathologist, using Scverse’s napari visualization tool to simultaneously examine cell annotations and corresponding H&E images, providing expert validation of annotation accuracy and biological plausibility.

### Patching and segmentation quality

H&E image patches of size 256 × 256 px with an overlap of 64 px were generated for each slide. Using the previously established cell annotations and the spatial alignment between ST and H&E data, masks for nuclei segmentation and cell type classification were created. The final cell labels used here were Epithelial, Blood_vessel, Fibroblast_Myofibroblast, Myeloid, B_Plasma, T_NK, Specialized, Melanocyte, and Other (the latter grouping Unknown, Stem_like, and Less10).

**Fig. 13:**
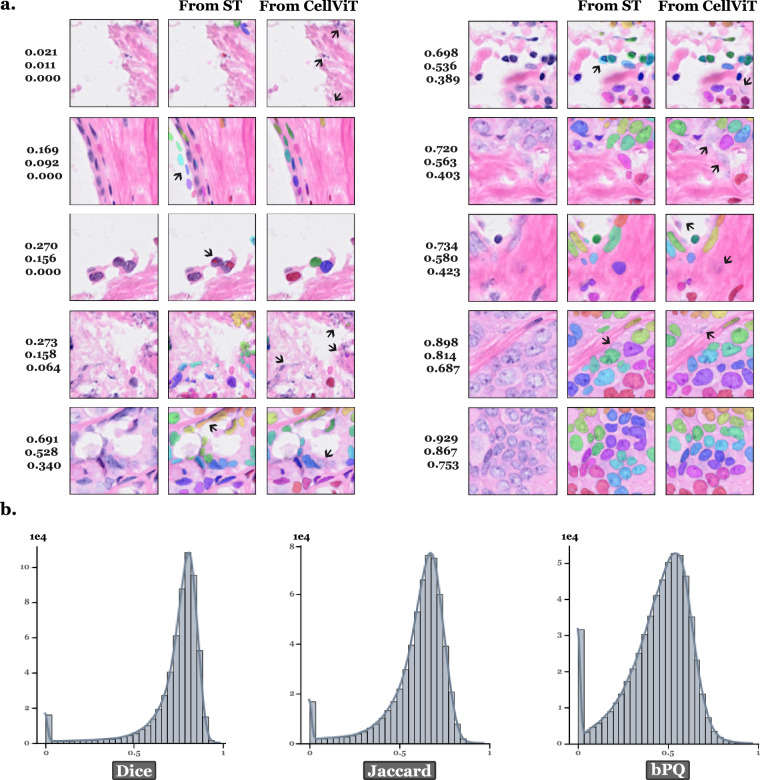
Pre-trained CellViT’s evaluation of the segmentation. The CellViT model pre-trained by the authors on the PanNuke dataset was applied to each slide. The predicted binary mask is compared with the segmentation mask coming from ST. The latter was generated using the segmentation on the DAPI and aligning the slide with the H&E modality. The comparison between the two masks for each patch is made using Dice coefficient, Jaccard index and bPQ scores. **a**. Examples of patches for breast_s0 with from left to right: H&E patch only, H&E patch with the mask from ST, and H&E patch with the mask generated by the pre-trained CellViT model. Patch scores are displayed at the left of each patch in the following order: Dice coefficient, Jaccard index and bPQ. Color identifies each unique nucleus and is not related to cell type here. Arrows highlight segmentation error examples. **b**. Distribution (bins = 30) of Dice, Jaccard, and bPQ scores for each patch over all the slides (brain_s0, bone_marrow_s0, bone_marrow_s1, bone_s0 excluded).

To evaluate the quality of segmentation and alignment, each generated mask was compared with segmentation predictions obtained from the pre-trained SAM-H/Hovernet CellViT model^7^. The Dice coefficient, Jaccard index, and Panoptic Quality (bPQ) metrics were employed for quantitative evaluation, with the resulting distributions across all patches (brain_s0, bone_marrow_s0, bone_marrow_s1, bone_s0 slides excluded) visualized in Fig. 13b.

The metric distributions demonstrate overall robust segmentation and alignment quality. To elucidate discrepancies between our segmentation and the CellViT predictions, representative patches illustrating both segmentation outputs for breast_s0 slide are presented in Fig. 13a. Each resulting score value corresponds to distinct scenarios. For instance, low scores may reflect regions with very few nuclei and they are undetected by one segmentation method, or significant misalignment between modalities. A low score can also correspond to poor image quality, for example due to blurring or other artifacts. Intermediate scores typically indicate minor alignment deviations or partial nuclei detection by either method. High scores generally indicate strong segmentation agreement, although minor detection discrepancies may persist. These segmentation quality scores have been incorporated into the dataset to facilitate dynamic filtering, allowing flexibility in selecting patches according to specific analytical requirements.

Importantly, as the Dice, Jaccard, and bPQ values quantify agreement with a pre-trained CellViT model, they should therefore be interpreted as model-dependent agreement scores rather than as absolute measures of mask correctness. Disagreement may arise not only from misregistration or segmentation errors in the Xenium-derived masks, but also from failure modes of the pre-trained CellViT model itself. This could be particularly important in tissue contexts that are underrepresented in the PanNuke training data or that exhibit atypical appearance, such as densely packed lymphoid regions, necrotic areas, mucus-rich tissue, or regions with strong staining variability and suboptimal focus. As a consequence, the QC procedure may also down-weight or remove morphologically challenging regions that are nonetheless well supported by the DAPI signal. Additionally, because CellViT is later fine-tuned on STHELAR, filtering patches based on agreement with a pre-trained CellViT model can introduce a mild inductive bias toward regions that are easier for CellViT-like architectures. We therefore emphasize that this QC step serves as a pragmatic filter for detecting extreme disagreement and likely technical artifacts, rather than as a gold-standard validation of nuclear segmentation.

**Fig. 14:**
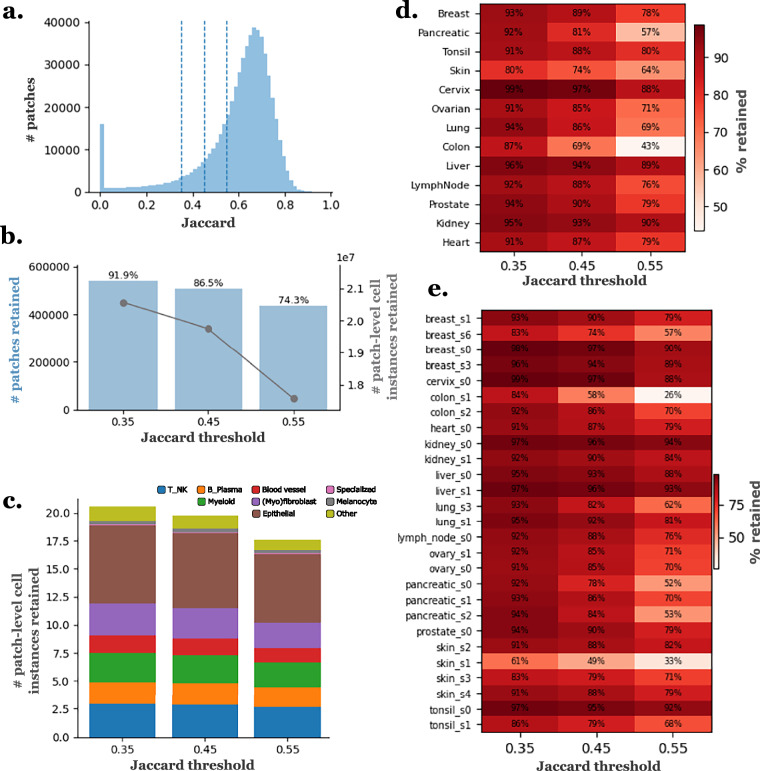
Sensitivity analysis of patch-level QC to the Jaccard threshold. Patch-level quality control compares Xenium-derived nuclei masks (from DAPI segmentation and ST-H&E co-registration) to masks predicted by a pre-trained CellViT model, and filters patches using a Jaccard threshold. **a**. Distribution (bins = 60) of Jaccard scores across all patches, with candidate thresholds (0.35, 0.45, 0.55) indicated. **b**. Effect of threshold on retained dataset size (number of patches) and on the total number of patch-level cell instances. **c**. Retained patch-level cell instances stratified by cell-type category for each threshold. **d**. Percentage of patches retained within each tissue for each threshold. **e**. Percentage of patches retained within each slide for each threshold.

To quantify the impact of the patch-level QC threshold on dataset composition, we evaluated three candidate Jaccard cutoffs (0.35, 0.45, and 0.55). Figure 14a shows the global distribution of Jaccard scores across all patches. As expected, increasing the threshold progressively removes patches from the low-agreement tail of this distribution. Figure 14b–e summarize how this choice affects the retained training corpus. Specifically, Fig. 14b reports the resulting number of retained patches and patch-level cell instances, Fig. 14c shows the corresponding cell-type composition, and Fig. 14d,e show retention rates stratified by tissue type and slide IDs, respectively. Across all three thresholds, the relative distributions of cell types, tissues, and slide identifiers remain largely conserved (Fig. 14c–e), indicating that the QC procedure primarily affects the overall dataset size rather than substantially reshaping its composition. Notably, patches with low agreement scores tend to cluster more strongly by slide identifier than by tissue type (Fig. 14d,e), suggesting that reduced agreement is driven in large part by slide-specific technical factors. This interpretation should, however, be viewed cautiously, as each tissue is represented by a limited number of slides.

### Dataset for fine-tuning the CellViT model

**Fig. 15:**
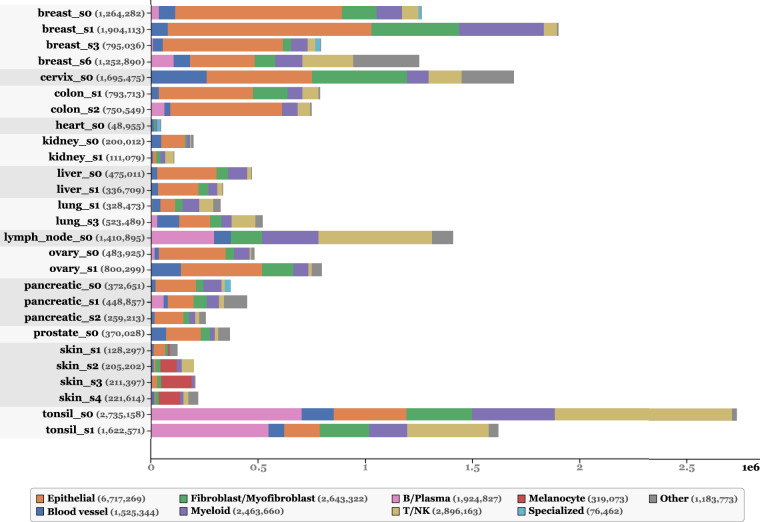
Patch-level nuclei instance counts for each slide in the dataset after making H&E patches and after filtering patches with a Jaccard index below 0.45. The total number of nuclei for each slide or for each cell type is shown in parentheses. The count is on a logarithmic scale. The same cell can be counted several times since it can be present on several patches (i.e., count is reported at the *patch-instance level*). Inspired by CellViT article^7^.

To demonstrate the utility of our dataset for downstream predictive tasks, we fine-tuned the CellViT model using STHELAR annotations. The patch dataset was processed following the CellViT authors’ data preparation pipeline, converting annotation masks into the input format required by the CellViT model. Given the large number of patches and categories, file storage formats were adapted for sparse representation to mitigate excessive storage requirements. A flexible pipeline was established to dynamically use various dataset configurations without requiring the reconstruction of data. Two distinct versions of the dataset were prepared for separate fine-tuning experiments. The first version employed detailed cell-type labels as described above, while the second adopted broader labels expected to facilitate more straightforward predictions. Detailed labels, such as distinguishing T lymphocytes from B lymphocytes, are often challenging due to their morphological similarities, which even experienced pathologists find difficult to differentiate. To get more generalized labels, specific cell categories were grouped as follows: Immune combined T_NK, B_Plasma, and Myeloid; Stromal merged Blood_vessel and Fibroblast_Myofibroblast; and Other grouped Specialized and the original Other category. Additionally, both dataset versions underwent quality filtering based on agreement with the pre-trained CellViT reference model, retaining only patches with a Jaccard score above 0.45 for the fine-tuning corpus. This resulted in a final selection of 508,483 patches at 40× from an original total of 587,555. Because the QC signal is derived from a CellViT model, this filtering step may preferentially remove morphologically difficult regions in addition to genuinely misregistered or low-quality patches. Consequently, the downstream fine-tuning results should be interpreted as conditional on this QC choice. To ensure transparency and reproducibility, all per-patch QC metrics are released, enabling users to apply alternative thresholds or retain all patches if desired (Fig. 14). The final dataset after patching and filtering is illustrated in Fig. 15. Importantly, two different “sizes” apply depending on the unit of counting: (i) *unique biological cells* ( ~11M, distinct Xenium cell IDs in the SpatialData), and (ii) *patch-level cell instances* (~19.7M, distinct cellID_patchID occurrences used by patch-based training files such as cell_count.csv and labels.zip). The second number is larger because tiling uses a 64 px overlap and because nuclei can be split across patch boundaries, causing the same biological cell to appear in multiple patches. This duplication increases the apparent instance count but does not create additional unique cells.

### CellViT fine-tuning

The CellViT architecture has been described in the original publication^7^. In summary, this model consists of a Vision Transformer (ViT) encoder connected via skip connections to four separate decoder modules. These decoders are organized into three distinct multitask output branches, each performing specific segmentation tasks. The first branch predicts binary segmentation maps for all nuclei (NP), delineating their precise boundaries and shapes. The second branch generates horizontal and vertical distance maps (HV), facilitating accurate localization and segmentation of individual nuclei. The third branch predicts nuclei type maps (NT) for classifying cells into distinct types. Postprocessing involves separating overlapping nuclei and assigning nuclei types through a majority voting mechanism applied within each nuclear region, based on the predicted NT map. Additionally, a tissue classification (TC) branch is implemented^7^.

The total loss function used to train the CellViT model is defined as:

1 $${L}_{total}={L}_{NP}+{L}_{HV}+{L}_{NT}+{L}_{TC}$$

where *L*_*NP*_ denotes the loss for the NP-branch, *L*_*HV*_ the loss for the HV-branch, *L*_*NT*_ the loss for the NT-branch, and *L*_*TC*_ the loss for the TC-branch. Each individual branch loss is formulated as a weighted combination of specific loss functions:

2 $$\begin{array}{rcl}{L}_{{\rm{NP}}} & = & {\lambda }_{{{\rm{NP}}}_{{\rm{FT}}}}{L}_{{\rm{FT}}}+{\lambda }_{{{\rm{NP}}}_{{\rm{DICE}}}}{L}_{{\rm{DICE}}}\\ {L}_{{\rm{HV}}} & = & {\lambda }_{{{\rm{HV}}}_{{\rm{MSE}}}}{L}_{{\rm{MSE}}}+{\lambda }_{{{\rm{HV}}}_{{\rm{MSGE}}}}{L}_{{\rm{MSGE}}}\\ {L}_{{\rm{NT}}} & = & {\lambda }_{{{\rm{NT}}}_{{\rm{FT}}}}{L}_{{\rm{FT}}}+{\lambda }_{{{\rm{NT}}}_{{\rm{DICE}}}}{L}_{{\rm{DICE}}}+{\lambda }_{{{\rm{NT}}}_{{\rm{BCE}}}}{L}_{{\rm{BCE}}}\\ {L}_{{\rm{TC}}} & = & {\lambda }_{{{\rm{TC}}}_{{\rm{CE}}}}{L}_{{\rm{CE}}}\end{array}$$

with *L*_*CE*_ the cross-entropy loss, *L*_*BCE*_ the binary cross-entropy loss, *L*_*DICE*_ the Dice loss, *L*_*FT*_ the Focal Tversky loss, *L*_*MSGE*_ the mean squared error of the gradients, and *L*_*MSE*_ the mean squared error.

The CellViT model^41^ was fine-tuned independently for each dataset configuration. For each configuration, the QC-filtered patch corpus (Jaccard  > 0.45) was split at the patch level into training (60%), validation (20%), and test (20%) subsets. Importantly, the split was performed within each slide, meaning that the same set of WSIs is present in the train, validation, and test sets. Because splitting was performed at the patch level rather than at the WSI level, WSI-level independence is not guaranteed, i.e., patches originating from the same slide appear in both the training and testing subsets. Consequently, the reported results should be interpreted as patch-level generalization within the same slide cohort, and may be optimistic compared to a strictly slide-disjoint (WSI-held-out) evaluation. This design choice was motivated by the limited number of available WSIs and the need to preserve representation of all tissues in each subset.

Fine-tuning was performed for the CellViT model variant using a Segment Anything Model (SAM)-based encoder and a HoVerNet-style decoder. The model architecture remained unchanged, except for the NT branch, where the final decoder layer was adapted to accommodate our custom set of cell type labels. Initialization was conducted using pre-trained weights from the CellViT-SAM-H model 40×, which employs a ViT-Huge encoder with 632 million parameters. This encoder includes 32 transformer blocks, 16 attention heads, and an embedding dimension of 1280.

The final fine-tuning did not use the authors’ original sampling strategy, as preliminary tests indicated that the class-weight strategy applied to the focal Tversky (FT) loss for the NT branch was sufficient. The loss weighting scheme used during training was consistent with the original configuration. Specifically, the TC branch used a weight of *λ* = 0.1 for the cross-entropy loss. The NP branch applied *λ* = 1.0 for both the Focal Tversky loss and Dice loss. The HV branch used *λ* = 2.5 for mean squared error (MSE) and *λ* = 8.0 for mean squared gradient error (MSGE). For the NT branch, *λ* = 0.5 was used for binary cross-entropy (BCE), *λ* = 0.2 for Dice loss, and *λ* = 0.5 for the multi-class Focal Tversky loss. Within the multi-class Focal Tversky loss, class weights were adjusted using the formula ${w}_{c}=\log (1+1/fre{q}_{c})$, where *freq*_*c*_ denotes the frequency of class *c* in the training set. The background weight was set equal to that of the most common cell type. As the Other category grouped cells with low RNA content or no clear marker genes, its contribution to the NT branch losses was downweighted to 0.1 for BCE and Dice loss, and to 0.1 times the weight of the most prevalent class for the Focal Tversky loss.

Training was performed for 53 or 42 epochs for detailed and grouped labels respectively, using mixed precision and a batch size of 32. The AdamW optimizer was employed with a learning rate of 3.10^−4^, a weight decay of 1.10^−4^, and *β* values of (0.85, 0.95). An exponential learning rate scheduler with a decay factor of *γ* = 0.85 was applied. Full model fine-tuning began from epoch 2 by unfreezing the encoder. Validation metrics were monitored throughout training, and the best models were selected based on these metrics: epoch 40 for the detailed label configuration, and epoch 32 for the grouped label configuration. Both fine-tuning experiments (detailed versus grouped label sets) were run using a fixed random seed. Running multiple independent fine-tuning runs with different seeds would substantially increase the computational cost of this study, given the size of the ViT-Huge encoder and the number of patches, and falls outside the scope of this Data Descriptor, which is primarily focused on dataset construction and release rather than exhaustive model benchmarking. The reported test-set metrics should therefore be interpreted as the outcome of a single training run per configuration. To provide transparency, we include the full training and validation loss curves for all CellViT branches, together with example per-class F1 detection curves, in supplementary information in Figure S1.

Despite the large number of cells, the current dataset originates from a limited number of slides. This constraint poses a challenge for the development of generalizable models, particularly when applied to WSIs coming from independent sources or different experimental conditions. To partially mitigate this limitation, the default data augmentation pipeline was retained during training, incorporating random rotations, flips, elastic deformations, blurring, Gaussian noise, and cropping. The only modification was the replacement of color jitter augmentation with a histology-specific HED color augmentation^42^. All images were normalized using mean and standard deviation values of (0.5, 0.5, 0.5).

Performance results on the held-out patch-level test set are summarized in Table 7 for detailed labels, and Table 8 for grouped labels. These results indicate that the CellViT model, originally trained on the PanNuke dataset, can effectively adapt to our Xenium-derived dataset through targeted fine-tuning. When trained on the nine detailed cell-type classes, the model retains excellent binary segmentation performance inherited from the pre-training (mean Dice = 0.883, mean Jaccard = 0.794, F1 = 0.855, and bPQ = 0.652). Despite nearly doubling the complexity of the label space and introducing morphologically challenging immune cell subtypes, the class-aware Panoptic Quality (mPQ) remains respectable at 0.369 (compared to 0.496 for initial CellViT performance on PanNuke). Performance is notably strong for morphologically distinct phenotypes, with Panoptic Quality (PQ) scores of 0.541 and 0.535 and F1 scores of 0.720 and 0.772 for epithelial cells and melanocytes, respectively. Classification is more challenging for inflammatory cell subtypes, yet performance remains encouraging, with PQ values ranging from 0.208 to 0.336 and F1 scores between 0.329 and 0.428. For the different connective cell types ((myo)fibroblast cells and blood vessels), PQ and F1 scores were 0.343 and between 0.404 and 0.490, respectively. To compare with pre-trained CellViT on PanNuke, the global inflammatory and connective categories achieve PQ values of 0.417 and 0.423, and F1 scores of 0.58 and 0.53, respectively. Cells categorized as Other have relatively low scores, which is expected given the intentionally reduced training weight assigned to this category. This decision reflects the inherent ambiguity and limited RNA content of these cells, allowing the model greater flexibility in classification. Segmentation quality is consistent across various tissues, indicated by Dice scores between 0.847 and 0.908 and bPQ values from 0.539 to 0.727.

**Table 7:** Evaluation on the held-out patch-level test set using CellViT fine-tuned with detailed cell type annotations.

Binary dataset metrics				
Binary-Cell-Dice-Mean		0.883		
Binary-Cell-Jaccard-Mean		0.794		
Tissue-Multiclass-Accuracy		0.999		
bPQ / bDQ / bSQ		0.652 / 0.820 / 0.792		
mPQ / mDQ / mSQ		0.369 / 0.462 / 0.572		
F1 / precision / recall (detection)		0.855 / 0.884 / 0.829		
**Tissue-specific metrics**				
Tissue	Dice	Jaccard	mPQ	bPQ
Breast	0.895	0.812	0.392	0.665
Cervix	0.889	0.802	0.375	0.671
Colon	0.847	0.737	0.316	0.539
Heart	0.867	0.768	0.317	0.644
Kidney	0.905	0.828	0.390	0.727
Liver	0.908	0.833	0.444	0.725
Lung	0.877	0.783	0.352	0.652
LymphNode	0.840	0.726	0.262	0.609
Ovarian	0.879	0.786	0.384	0.656
Pancreatic	0.863	0.762	0.335	0.596
Prostate	0.859	0.755	0.378	0.630
Skin	0.874	0.780	0.390	0.640
Tonsil	0.865	0.763	0.257	0.630
**Nuclei type metrics**				
Nuclei Type		DQ	SQ	PQ
T_NK		0.409	0.544	0.336
B_Plasma		0.259	0.450	0.208
Myeloid		0.370	0.547	0.294
Blood_vessel		0.441	0.537	0.343
Fibroblast_Myofibroblast		0.438	0.595	0.343
Epithelial		0.664	0.732	0.541
Specialized		0.242	0.300	0.195
Melanocyte		0.652	0.703	0.535
Other		0.228	0.346	0.170
**Nuclei detection metrics**				
Nuclei Type		Precision	Recall	F1
T_NK		0.401	0.460	0.428
B_Plasma		0.428	0.365	0.394
Myeloid		0.367	0.298	0.329
Blood_vessel		0.559	0.437	0.490
Fibroblast_Myofibroblast		0.422	0.387	0.404
Epithelial		0.705	0.736	0.720
Specialized		0.580	0.282	0.379
Melanocyte		0.791	0.755	0.772
Other		0.453	0.305	0.365

**Table 8:** Evaluation on the held-out patch-level test set using CellViT fine-tuned with grouped cell type annotations.

Binary dataset metrics				
Binary-Cell-Dice-Mean		0.885		
Binary-Cell-Jaccard-Mean		0.796		
Tissue-Multiclass-Accuracy		0.999		
bPQ / bDQ / bSQ		0.656 / 0.823 / 0.794		
mPQ / mDQ / mSQ		0.421 / 0.527 / 0.632		
f1 / precision / recall (detection)		0.859 / 0.890 / 0.830		
**Tissue-specific metrics**				
Tissue	Dice	Jaccard	mPQ	bPQ
Breast	0.896	0.814	0.441	0.669
Cervix	0.891	0.805	0.413	0.677
Colon	0.848	0.738	0.365	0.543
Heart	0.865	0.766	0.388	0.645
Kidney	0.903	0.825	0.419	0.722
Liver	0.909	0.834	0.508	0.728
Lung	0.877	0.783	0.411	0.656
LymphNode	0.843	0.730	0.325	0.612
Ovarian	0.880	0.788	0.424	0.659
Pancreatic	0.866	0.767	0.390	0.605
Prostate	0.859	0.755	0.417	0.630
Skin	0.876	0.784	0.424	0.642
Tonsil	0.867	0.767	0.355	0.635
**Nuclei type metrics**				
Nuclei Type		DQ	SQ	PQ
Immune		0.492	0.628	0.395
Stromal		0.527	0.666	0.413
Epithelial		0.663	0.730	0.543
Melanocyte		0.665	0.712	0.548
Other		0.216	0.322	0.163
**Nuclei detection metrics**				
Nuclei Type		Precision	Recall	F1
Immune		0.626	0.625	0.625
Stromal		0.561	0.482	0.518
Epithelial		0.720	0.732	0.726
Melanocyte		0.798	0.766	0.782
Other		0.490	0.282	0.358

When closely related phenotypes, difficult to distinguish based only on H&E morphology, were merged into five broader classes, classification metrics improved significantly without compromising segmentation performance. In comparison with the pre-trained CellViT results, the fine-tuned model achieved PQ scores of 0.395 for immune cells (versus 0.417 for inflammatory for the pre-trained), 0.413 for stromal cells (versus 0.423 for connective for the pre-trained), and 0.543 and 0.548 for epithelial and melanocyte categories (versus 0.581 and 0.583 for neoplastic and epithelial in the pre-trained case). Correspondingly, F1 scores improved to 0.625 for immune (versus 0.58 for inflammatory for the pre-trained), 0.518 for stromal (versus 0.53 for connective for the pre-trained), and 0.726 and 0.782 for epithelial and melanocyte categories (versus 0.71 and 0.73 for neoplastic and epithelial in the pre-trained case).

These performance metrics, particularly PQ metrics, can be impacted by the accuracy of segmentation. Given that our ground-truth segmentation may exhibit minor pixel-level inaccuracies due to slight misalignments, we further investigated model performance using a confusion matrix within the matched pairs prediction/ground-truth nuclei obtained through the Kuhn-Munkres algorithm (Table 9). This analysis confirms overall agreement. As expected, it is more difficult to differentiate B and T lymphocytes that are nearly indistinguishable morphologically in H&E. Future improvements might be to restrict the test set to patches with the highest segmentation quality scores. This approach could ensure greater confidence in performance metrics by minimizing the confounding effects of segmentation inaccuracies.

**Table 9:** Cell type classification confusion matrix on the test set.

True type	Predicted type									
	Background	T/NK	B/Plasma	Myeloid	Blood vessel	(Myo)Fibroblast	Epithelial	Specialized	Melanocyte	Other
T/NK	0.01	**0.69**	0.11	0.08	0.01	0.05	0.03	<0.01	<0.01	0.02
B/Plasma	0.01	0.25	**0.58**	0.05	0.01	0.05	0.03	<0.01	<0.01	0.01
Myeloid	0.01	0.14	0.05	**0.50**	0.03	0.12	0.13	<0.01	<0.01	0.02
Blood vessel	0.01	0.04	0.01	0.06	**0.67**	0.13	0.05	<0.01	<0.01	0.02
(Myo)Fibroblast	0.01	0.10	0.05	0.10	0.05	**0.62**	0.05	<0.01	<0.01	0.02
Epithelial	<0.01	0.01	0.01	0.02	0.01	0.01	**0.94**	<0.01	<0.01	0.01
Specialized	0.01	0.01	0.01	0.05	0.06	0.04	0.34	**0.48**	0	<0.01
Melanocyte	<0.01	0.01	<0.01	0.02	<0.01	0.01	0.01	0	**0.94**	<0.01
Other	0.02	0.12	0.02	0.07	0.05	0.10	0.07	<0.01	<0.01	**0.55**

The confusion matrix compares ground truth labels and predictions from the CellViT model fine-tuned on detailed cell types. The comparison is performed only within the matched pairs of ground-truth/predicted nuclei obtained using the Kuhn-Munkres algorithm.

An increase in the number of available slides would also make it possible to improve the overall segmentation quality of the dataset. With a larger dataset, we could apply more stringent filtering criteria during patch selection, retaining only regions with excellent segmentation and accurate alignment between modalities. This selective approach would raise the average quality of the final dataset. In parallel, improving the criteria used for patch selection may further enhance consistency. For instance, switching to a selection using the PQ metric, which simultaneously accounts for segmentation and classification performance, could provide a more comprehensive and quantitative basis for patch evaluation. Additionally, using more advanced alignment tools, such as the Valis method, could help correct residual spatial misalignments in certain patches. A combined approach that leverages stricter selection, better metrics, and improved alignment techniques may offer the most robust solution. Interestingly, since the pre-trained CellViT’s predictions have been computed for each patch, the dataset may also be used to evaluate modality realignment methods, highlighting an additional potential application.

### PanNuke labels

To assess the cell type prediction performance of pre-trained CellViT, the model was applied to our dataset in inference mode. The architecture used was consistently the SAM-H/HoVerNet variant of CellViT, pre-trained on PanNuke. To ensure a direct cell comparison between predicted cell types and those obtained through RNA-based clustering, predicted labels from CellViT output were not used directly. Instead, pixel-level probability maps for each cell type were retrieved from the NT branch output, and the post-processing was implemented using Xenium-derived segmentation masks. Multiple evaluation strategies were applied, including confusion matrices, Sankey diagrams, and UMAP visualizations. These analyses were performed at both the full dataset level and on a slide-by-slide basis to explore prediction consistency and discrepancies.

The same analysis pipeline was applied to the outputs of our fine-tuned CellViT models, primarily for exploratory purposes. These comparisons offer insight into persistent sources of confusion between cell types, but interpretation remains limited by the fact that cells are not coming only from the evaluation set but also from training and validation sets.

Table 10 provides an overview of the results for the pre-trained CellViT model. Myeloid cells seem to be particularly challenging to predict, as they are frequently misclassified as connective instead of inflammatory. Furthermore, contrary to expectations, cells with fewer than 10 RNAs are rarely classified as dead cells, potentially highlighting a limitation in either the prediction capability or the underlying assumption that low RNA content directly indicates cell death. Distinguishing between normal epithelial and neoplastic cells also seems problematic. While a predominant classification of epithelial cells as neoplastic is reasonable considering the number of cancerous tissues, some normal epithelial cells should still be expected. For instance, the slide liver_s0 is derived from a healthy patient, but it exhibits a 64% classification rate of epithelial cells as neoplastic, highlighting this limitation. It should also be mentioned that heart, tonsil, and lymph node tissues are present in our dataset but absent from the original PanNuke dataset, potentially affecting model generalization and accuracy for these specific tissues.

**Table 10:** Comparison between RNA-based cell type annotations and PanNuke labels predicted by the pre-trained CellViT model (row-normalized).

Cell type	PanNuke label					
	Background	Connective	Dead	Epithelial	Inflammatory	Neoplastic
B_Plasma	0.05	0.12	0	0.01	**0.60**	0.21
Blood_vessel	0.12	**0.68**	0	0.01	0.11	0.09
Epithelial	0.10	0.24	0	0.03	0.03	**0.60**
Fibroblast_Myofibroblast	0.12	**0.62**	0	0.01	0.16	0.09
Less10	0.30	**0.42**	0.01	0.01	0.18	0.07
Melanocyte	0.12	0.11	0	0.02	0.01	**0.75**
Myeloid	0.11	**0.47**	0	0.01	0.23	0.18
Specialized	0.10	**0.56**	0	0.01	0.04	0.29
Stem_like	0.18	**0.56**	0	0.01	0.09	0.16
T_NK	0.04	0.20	0	0.01	**0.60**	0.15
Unknown	0.19	**0.46**	0.01	0.01	0.23	0.09

Each row corresponds to a cell type defined using transcript information. Columns indicate the proportion of cells assigned to each PanNuke category by the CellViT model, using Xenium-derived segmentation masks.

Overall, we can see that the broad categorization employed by PanNuke inherently results in loss of detailed cellular information. Hence, exploring approaches for more refined predictions appears promising for better characterizing cell types.

### H&E features

To compare cell-level embeddings derived from H&E images with those obtained from RNA data, three different encoders were applied to the H&E patches for the whole dataset. In addition to the pre-trained CellViT encoder, two other pre-trained ViT were evaluated. The first was the Phikon-v2 model, developed by Owkin, which is a ViT Large pre-trained using the DinoV2 self-supervised learning approach on the PANCAN-XL dataset^43^. PANCAN-XL comprises approximately 450 million histology image tiles at 20× magnification, sampled from over 60,000 WSIs. The second model was Google’s ViT-Base-Patch16-224-in21k, a transformer encoder (BERT-like architecture) trained in a supervised manner on the ImageNet-21k dataset^44^. For all three models, cell-level embeddings were extracted as follows: for each cell in a given patch, sub-patches containing at least one pixel belonging to that cell were identified. In this context, “sub-patches” refer to the input tokens used by transformer architectures. Embeddings were extracted for each relevant sub-patch and then averaged to produce a single patch-level embedding per cell. When a cell appeared in multiple patches, the embeddings from those patches were further averaged to generate a single global embedding per cell. For CellViT, nucleus segmentation was used to define relevant sub-patches, while for the Phikon-v2 and Google models, cell segmentation boundaries were used. For each model and each slide, PCA was performed on the resulting cell embeddings. We retained the minimum number of principal components required to explain 95% of the total variance, and used these components as input for k-means clustering. The optimal number of clusters was determined using the Elbow method combined with the KneeLocator algorithm. Cluster assignments were visualized on the UMAP projections of the RNA-derived data, allowing for direct comparison of clustering patterns between the two modalities. This whole process was repeated using the fine-tuned CellViT models to assess whether fine-tuning improved alignment between H&E-derived and RNA-derived embeddings, particularly for the model fine-tuned with more detailed cell type labels. However, as with earlier comparisons, it is important to interpret these results cautiously, as the features were extracted from all cells and not exclusively from the test set.

**Fig. 16:**
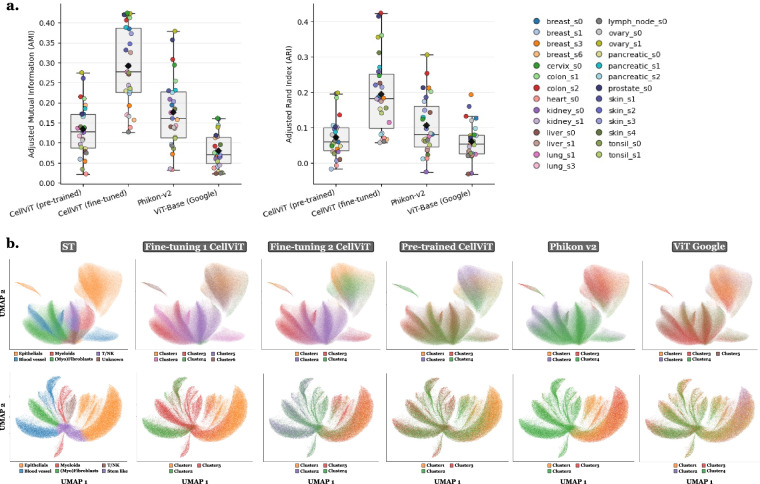
Comparison of H&E-derived cell features and RNA-derived final cell-type labels across WSI encoders. **a**. Quantitative agreement between H&E-embedding k-means clusters and RNA-derived final cell-type labels across encoders. For each slide and each H&E encoder, we clustered per-cell H&E embeddings using k-means and compared the resulting cluster assignments to the RNA-derived final labels using Adjusted Mutual Information (AMI) and Adjusted Rand Index (ARI). Each dot corresponds to one slide (colored by slide ID). Boxplots summarize the distribution across slides with black diamonds indicating the mean. For the fine-tuned CellViT encoder, results are given only for the fine-tuning on the detailed cell types. **b**. Visual comparison of H&E-derived features for slides cervix_s0 (top) and prostate_s0 (bottom). UMAPs are based on RNA-derived embeddings used during the cell type annotation process. Cluster colors (left to right) represent: (1) cell types assigned from RNA-based labeling, k-means clusters derived from nucleus features extracted using (2) CellViT fine-tuned on detailed cell types, (3) CellViT fine-tuned on grouped cell types, (4) pre-trained CellViT, and k-means clusters derived from cell features extracted using (5) Phikon-v2 encoder, and (6) ViT-Base encoder from Google.

We quantified cross-modal agreement between H&E-derived unsupervised structure and the RNA-derived final cell-type labels. Specifically, for each slide and each encoder, we computed Adjusted Mutual Information (AMI) and Adjusted Rand Index (ARI) between the k-means cluster assignment obtained from H&E embeddings and the final RNA-derived cell-type label for the same cell IDs (Fig. 16a). Across the 27 slides included in this comparison, fine-tuning CellViT yields a clear improvement: the median AMI increases from 0.127 (pre-trained) to 0.277 (fine-tuned), and the median ARI increases from 0.060 to 0.181 (means: AMI 0.134 to 0.292; ARI 0.072 to 0.194). Phikon-v2 shows intermediate agreement (median AMI 0.161; median ARI 0.081), while ViT-Base-Google shows the lowest agreement overall (median AMI 0.070; median ARI 0.052). Representative examples of feature evolution are shown in Fig. 16b, where RNA-based embeddings are projected using UMAP and colored by k-means cluster assignments derived from each H&E encoder. Visual alignment is considered better when these clusters correspond to well-separated regions in the RNA-derived space. Although agreement remains moderate, as expected because H&E morphology is only an indirect proxy of transcriptionally defined identity and because labels were curated at a coarse pan-cancer resolution, these results provide quantitative evidence that adapting the CellViT encoder to STHELAR increases alignment between histology-derived embeddings and RNA-derived cell-type structure. This improvement is not consistently observed across all slides, which is not surprising given the diversity and complexity of the data. Moreover, improved clustering alignment does not necessarily predict enhanced performance in subsequent tasks, although these results remain insightful.

An alternative approach to mitigate the dependency on segmentation quality would be to use the dataset on the recently proposed CellViT++^41^. In this pipeline, CellViT is first applied to extract both segmentation masks and feature embeddings for each nucleus. These embeddings can then be used as input for training a separate classifier, allowing the prediction of other cell types. This strategy offers a promising path for leveraging the dataset’s biological content, particularly in cases where segmentation may not be perfectly aligned between modalities, even if a baseline level of segmentation quality remains essential to ensure correspondence between the segmentation masks in our dataset and those produced by CellViT. Nevertheless, this decoupling has limitations since the CellViT++ solution does not enable fine-tuning the encoder. As a result, if the pre-trained features lack the discriminative information required to distinguish between specific cell types, their classification by the classifier will be limited. Our experiments comparing nucleus-level features using k-means clustering before and after CellViT encoder fine-tuning suggest that adjusting the feature space could be relevant for more precise cell type classification.

### Cell-type prediction from frozen pre-trained CellViT embeddings

Assessing generalizability would ideally involve training and evaluating in cross-slide and cross-tissue settings. However, training the full CellViT model is computationally demanding and time-intensive. To obtain an initial estimate of how well histology-derived representations and cell-type predictions transfer across sections and tissues, without retraining the complete CellViT model, we adopted the following compromise: we trained a lightweight classifier directly on the precomputed pre-trained CellViT per-cell embeddings. This approach is inspired by the CellViT++ design^41^. As discussed in the previous paragraph, because the encoder may change when adapting to the expanded set of cell types, relying on the fixed pre-trained encoder can limit performance. Nevertheless, this lightweight classifier provides a first approximation and enables preliminary conclusions about cross-section and cross-tissue transfer.

For each Xenium slide, we used (i) the per-cell embedding vector produced by the pre-trained CellViT encoder in the previous H&E features paragraph and stored in tables/features_cellvit in each SpatialData object, (ii) the Xenium-derived cell-type annotation (i.e., final labels using whole-cell RNA counts with the following categories: Epithelial, Blood_vessel, Fibroblast_Myofibroblast, Myeloid, B_Plasma, T_NK, Melanocyte, Other, where Other includes here Less10, Unknown, Stem_like, and Specialized labels). We evaluated cell-type prediction under multiple cross-validation (CV) splitting regimes designed to analyze cross-slides, cross-tissues, within-tissues, and within-slides configurations.

Before any split was applied, we filtered cells using two global quality criteria, applied identically to train / validation / test in every fold. First, we removed cells with within-slide RNA-depth quantile below min_rna_depth_quantile = 0.02. Second, we assigned to each cell a segmentation/alignment score by taking the Jaccard mean of patches that the given cell belongs to. Then we removed cells with a Jaccard_mean  <0.2. These filters reduced the influence of low-RNA cells and low-confidence segmentations on downstream evaluation. We evaluated four complementary regimes. (i) **Cross-slide CV** (cross_slides): generalization across WSIs was assessed with an outer K-fold split over slides (outer_folds = 5). In each outer fold, held-out slides formed the test set. From the remaining slides (train+val pool), an inner K-fold split (val_folds = 5) selected a validation set at the slide level, with the inner fold choice deterministically rotated across outer folds. (ii) **Cross-tissue evaluation** (cross_tissues, leave-one-tissue-out): all slides from one tissue were held out as the test set, while the remaining tissues formed the train+val pool. The validation set was chosen as all slides from one entire tissue (randomly permuted in a deterministic, seed-controlled manner) to prevent leakage across tissues between train and validation. (iii) **Within-tissue, cross-slide CV** (within_tissues): for tissues with sufficient replication (minimum min_slides_per_tissue = 3, i.e. breast, pancreatic, and skin tissues), we performed slide-level CV within each tissue independently by running the same cross-slide procedure as above (outer_folds = 4, val_folds = 3, each capped by the tissue’s number of slides) using a tissue-specific seed derived deterministically from the global seed. (iv) **Within-slide CV** (within_slides, cell-level splitting): as a control setting that removes slide/tissue domain shift, we evaluated generalization within each individual slide by splitting cells rather than slides. A subset of 10 slides was selected to maximize tissue diversity by preferentially choosing one slide per tissue. For each selected slide, we applied K-fold splitting over cells (outer_folds = 5) to define held-out test cells, and then randomly split the remaining cells into train/validation with a fixed validation fraction (inner_val_frac = 0.2). Across all regimes, split membership was defined at the slide or cell level as described above, and all remaining preprocessing (label mapping and quality filters) was performed identically.

**Fig. 17:**
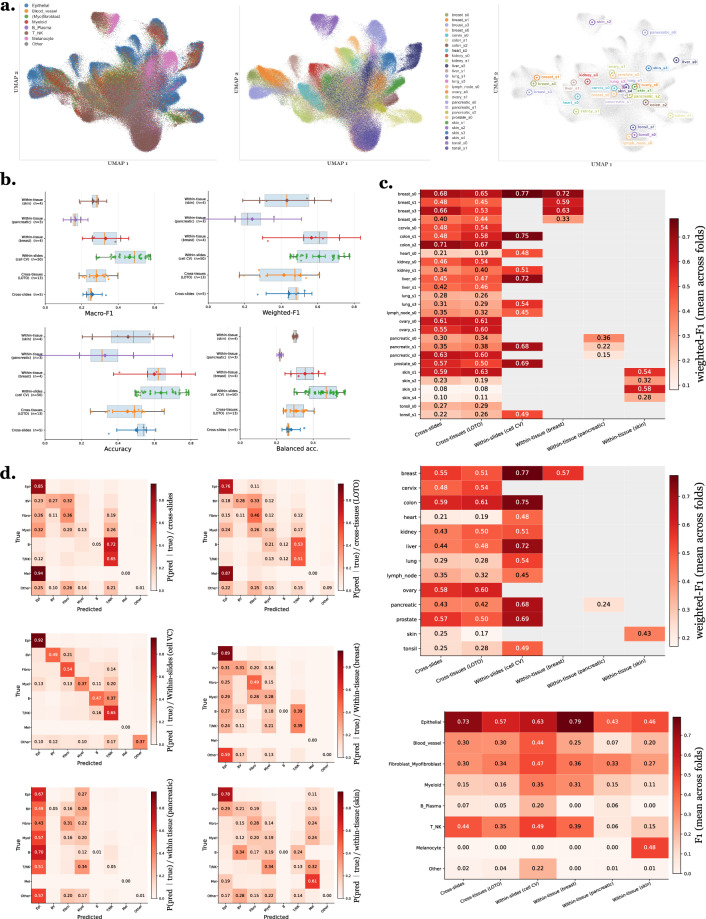
Cell-type prediction from frozen pre-trained CellViT embeddings across several split configurations. **a**. UMAP visualization of pre-trained CellViT per-cell embeddings after PCA reduction, shown (i) colored by grouped Xenium cell type (from final_label using whole-cell RNA counts), (ii) colored by slide_id, and (iii) as a grey background with per-slide centroids and labels overlaid to highlight slide-specific structure. **b**. Cross-validation performance distributions (one point per fold) for macro-F1, weighted-F1, accuracy and balanced accuracy across the different split configurations; diamonds and horizontal bars indicate the mean and its 95% confidence interval across folds. **c**. Breakdown of weighted-F1 averaged across folds, reported (top) per slide and (middle) per tissue; (bottom) per-class F1 averaged across folds. **d**. Mean row-normalized confusion matrices (aggregated across folds) for each split configuration, reporting $P(\widehat{y}| y)$ with rows as true classes and columns as predicted classes.

We trained a little classifier on frozen embeddings using the same hyperparameters in all regimes. All hyperparameters were fixed a priori, and no hyperparameter tuning was performed across regimes. The model was a linear head consisting of LayerNorm  → Dropout(0.2)  → Linear(D → C), where *D* is the embedding dimension and *C* is the number of classes. Training used AdamW optimization (lr = 3 × 10^−5^, weight_decay = 1 × 10^−4^) with a cosine learning-rate schedule (min_lr = 1 × 10^−6^, warmup_epochs = 2) for up to max_epochs = 20. The loss was multi-class cross-entropy with label smoothing (label_smoothing = 0.05). Gradient norms were clipped (grad_clip_norm = 1.0). Early stopping monitored validation macro-F1 (monitor = val_macro_f1, patience = 4 or 6, min_delta = 0.001), with validation evaluated every epoch. Figure 17b–d summarizes the results on the test sets. Panel b shows fold-wise distributions for F1, weighted-F1, accuracy, and balanced accuracy. Panel c reports mean performance across folds stratified by slide (Weighted-F1), tissue (Weighted-F1), and cell type (per-class F1). Panel d shows class confusions by summing confusion matrices across folds and row-normalizing the aggregate.

To complement the linear-probe evaluation on frozen CellViT embeddings, we generated also a visualization of the embedding space using all slides together. We reused the pre-trained CellViT per-cell embeddings and their associated per-cell metadata, applying the same global quality filters as in the classifier experiments (i.e., removing cells with RNA-depth quantile below 0.02 in cells_rna_depth_quantile and low-quality segmentations with Jaccard_mean <0.2). From each slide we then sampled up to 10, 000 filtered cells using a deterministic slide-specific seed, concatenated all sampled embeddings, projected them with PCA to 90 components, and computed a two-dimensional UMAP embedding (*n*_neighbors_ = 30, ${\min }_{{\rm{dist}}}=0.3$). The same UMAP coordinates were displayed using three complementary colorings: grouped cell types, slide identity, and a grey background view in which we overlaid the centroid of each slide (mean UMAP coordinate) and its label to expose slide-level clustering patterns. Figure 17a illustrates the results.

Across split configurations (Fig. 17b), within-slide evaluation provided the most favorable setting: when training and testing on different cell subsets from the same slide (cell-level CV), the linear head achieved macro-F1 0.463 (95% CI: [0.436, 0.490]), weighted-F1 0.608 ([0.574, 0.642]), accuracy 0.636 ([0.604, 0.668]), and balanced accuracy 0.454 ([0.430, 0.478]). In contrast, performance decreased when generalizing across acquisition units: in cross-slide CV, macro-F1 dropped to 0.259 ([0.207, 0.312]) and weighted-F1 to 0.443 ([0.317, 0.569]), while in leave-one-tissue-out evaluation macro-F1 was 0.283 ([0.243, 0.322]) and weighted-F1 0.415 ([0.325, 0.506]). Within-tissue cross-slide performance depended strongly on tissue, with breast exceeding skin and pancreatic in macro-F1 (breast 0.331; skin 0.262; pancreatic 0.166).

Per-class F1 (Fig. 17c) highlighted pronounced class-dependent behavior. Epithelial cells were consistently the most predictable, while stromal and immune categories were substantially harder, especially under cross-slide or cross-tissue generalization. For example, Blood_vessel and Fibroblast_Myofibroblast achieved moderate F1 that improved markedly within-slide (Blood_vessel 0.44, Fibroblast_Myofibroblast 0.47) relative to cross-slide/cross-tissue settings. Myeloid and B_Plasma remained challenging overall (e.g. B_Plasma near zero in several settings), and the Other group showed low F1 across all regimes, consistent with its heterogeneous definition. Melanocyte performance was essentially null except in skin (F1 0.48 within-tissue skin), reflecting that Melanocyte is tissue-restricted and/or absent from many folds after filtering.

Error structure in the aggregated confusion matrices (Fig. 17d) was coherent with these per-class trends. Across regimes, confusions were concentrated among biologically and morphologically proximal compartments: vascular and fibroblast-like classes frequently mixed, myeloid cells were often misclassified as epithelial/fibroblast/T_NK depending on regime, and B_Plasma exhibited strong confusion with T_NK (e.g., in cross-slide and cross-tissue settings), indicative of limited discriminability of these immune subtypes from frozen H&E embeddings alone. Importantly, the gap between within-slide and cross-slide/cross-tissue results, together with the slide-centric structure visible in the UMAP (Fig. 17a), supports the presence of substantial slide- and tissue-associated variation in the embedding space (e.g., staining, acquisition, or tissue morphology effects), which degrades out-of-domain generalization for a simple linear probe. Overall, these results validate that pre-trained CellViT embeddings contain a strong signal for broad compartments (notably epithelium) while also delineating the limits of separability for rarer and phenotypically overlapping immune cell types under realistic cross-slide and cross-tissue transfer.

## Supplementary information

Supplementary Information

## Data Availability

The STHELAR dataset is publicly available in the BioStudies database under accession **S-BIAD2146** at 10.6019/S-BIAD2146. This record contains the complete data release, including one SpatialData object per slide, per-cell metadata tables, and the H&E patch datasets at 40× and 20× with corresponding segmentation/classification masks and CellViT fine-tuning materials. For convenience, patch-only subsets with cell_id_map masks and associated per-slide metadata are also available on HuggingFace at 10.57967/hf/6008 (40×) and 10.57967/hf/6009 (20×).

## Code availability

Source code is available on GitHub in the two following repositories:

**STHELAR**: Code for generating the dataset and validation analysis, accessible at https://github.com/MICS-Lab/STHELAR.

**STHELAR_for_CellViT**: Code from the CellViT repository^45^ and adapted for our dataset, accessible at https://github.com/MICS-Lab/CellViT_for_STHELAR.
