# Supplementary material for: STHELAR, a multi-tissue dataset linking spatial transcriptomics and histology for cell type annotation
Source: Sci Data. 2026 Mar 12;13:665. doi: 10.1038/s41597-026-06937-6 (PMC13121762; doi:10.1038/s41597-026-06937-6)
Supplement: Supplementary file 1 — Supplementary Information [file 41597_2026_6937_MOESM1_ESM.pdf]

Supplementary information

STHELAR, a multi-tissue dataset linking spatial transcriptomics and histology for cell type annotation

Félicie Giraud-Sauveur[1,2,3] Quentin Blampey[1,2] Hakim Benkirane[1,2]
Arianna Marinello[4] Paul-Henry Cournède[1,2,5] Stergios Christodoulidis[1,2,6]

January 2026

Contents

1 Supplementary figure **2**
1.1 Training and validation curves for CellViT fine-tuning on STHELAR 2

[1]Paris-Saclay University, CentraleSupelec, Laboratory of Mathematics and Computer Science (MICS), Gif-sur-Yvette, France
[2]IHU PRISM: National Center for Precision Medicine in Oncology, Gustave Roussy, Villejuif, France
[3]felicie.giraud-sauveur@centralesupelec.fr
[4]Department of Medical Oncology, Gustave Roussy, International Center for Thoracic Cancers, Villejuif, France
[5]paul-henry.cournede@centralesupelec.fr
[6]stergios.christodoulidis@centralesupelec.fr

1 Supplementary figure

1.1 Training and validation curves for CellViT fine-tuning on STHELAR

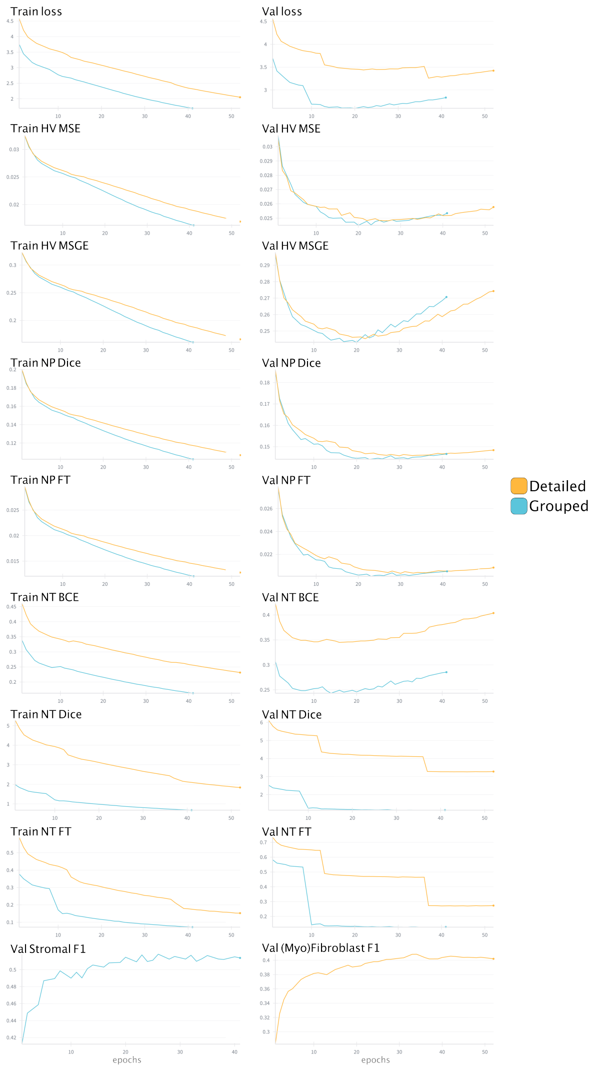

Figure S1: **Training and validation curves for CellViT fine-tuning on STHELAR.** The panels summarize the optimisation behaviour of the CellViT model fine-tuned on the STHELAR dataset for the two label configurations (detailed versus grouped). For each branch of the model, it shows the corresponding loss curves over training epochs, including both training and validation curves. Specifically, it reports: (i) HV-branch losses (mean squared error "HV MSE" and mean squared gradient error "HV MSGE"), (ii) NP-branch losses (Dice "NP Dice" and Focal Tversky "NP FT"), and (iii) NT-branch losses (binary cross-entropy "NT BCE", Dice "NT Dice", and multi-class Focal Tversky "NT FT"). In each panel, the curves for the fine-tuning with detailed cell-type labels are shown in yellow, and the curves for the fine-tuning with grouped labels are shown in blue. Two additional panels illustrate example validation F1 detection curves over epochs for (i) the `Stromal` class in the grouped-label configuration and (ii) the `(Myo)Fibroblast` class in the detailed-label configuration.
